# Evidence of endothelial anergy and T-cell exclusion in microsatellite stable colorectal liver metastases revealed through human hemi-liver perfusion and spatial transcriptomics

**DOI:** 10.1186/s12943-026-02732-4

**Published:** 2026-07-20

**Authors:** Peter Kok-Ting Wan, Ranchu Cheng, David Johnson, Carl Lee, Shihong Wu, Areeb Mian, Ahmet Hazini, Sorayya Moradi, Kate Friesen, Flurin Caviezel, Syed Hussain Abbas, Brannon Nicholls, Hatem Sadik, Adil Lakha, Keaton Jones, Girishkumar Kumaran, Matthew J. Bottomley, Rob Jones, Mark Coles, Rachael Bashford-Rogers, Constantin Coussios, Len Seymour, Robert Carlisle, Kerry Fisher, Alex Gordon-Weeks

**Affiliations:** 1 Department of Oncology, University of Oxford, Oxford, UK; 2 Nuffield Department of Surgical Sciences, University of Oxford, Oxford, UK; 3 Department of Engineering Science, Institute of Biomedical Engineering, University of Oxford, Oxford, UK; 4 Kennedy Institute of Rheumatology, University of Oxford, Oxford, UK; 5 Department of Biochemistry, University of Oxford, South Parks Road, Oxford, UK; 6 Nuffield Department of Medicine, Chinese Academy of Medical Sciences Oxford Institute, University of Oxford, Oxford, UK; 7 Department of Molecular and Clinical Cancer Medicine, University of Liverpool, Liverpool, UK

**Keywords:** Colorectal cancer liver metastasis, Human cancer models, Liver perfusion, Eendothelial anergy, T cell exhaustion, Spatial transcriptomics, Multiplexed imaging

## Abstract

**Background:**

Colorectal liver metastasis (CRLM) is the terminal stage of colorectal cancer and remains largely resistant to immunotherapeutics, yet the underlying immune escape mechanisms remain poorly understood. Through novel observational methods focused on human living and fixed tissue, we profiled the spatial and molecular features of microsatellite stable (MSS) CRLM and the surrounding liver microenvironment.

**Methods:**

First, we re-purposed normothermic hepatic machine perfusion as a preclinical model of MSS CRLM, enabling delivery of labelled T-cells through physiological vascular routes into living, tumour-bearing human livers. Second, to dissect the liver microenvironment, we spatially map the transcriptomes of MSS CRLM and adjacent liver at single-cell resolution across ~ 1.9 million cells, and perform multiplexed immunofluorescence applying a19-marker panel to a validation cohort.

**Results:**

Whilst T-cells readily extravasated within the peri-tumoural liver, they failed to extravasate into MSS CRLM. Spatial analyses revealed a progressive shift in endothelial phenotypes across regions: from a non-inflamed state in normal liver, to a highly inflamed, peri-tumoural endothelium, and finally to an anergic, angiogenic endothelial population within CRLM characterised by reduced expression of adhesion molecules required for T-cell extravasation. CD4 T-cells that extravasate in the peri-tumoural liver are TCR-reactive yet exhausted, whereas CD4 T-cells within CRLM show stress responses, impaired cytokine gene expression and reduced TCR reactivity, residing within hypoxic niches. These divergent CD4 phenotypes are associated with distinct fibroblast-myeloid niches across peri-tumoural and tumour regions.

**Conclusion:**

Based on these observations, MSS CRLM impair T-cell extravasation and simultaneously drive intra-tumoural endothelial anergy, peri-tumoural T-cell exhaustion and an intra-tumoural T-cell stress response. Further mechanistic work is needed to validate these findings and guide the development of novel immunotherapeutics.

**Supplementary Information:**

The online version contains supplementary material available at https://doi.org/10.1186/s12943-026-02732-4.

## Introduction

Liver metastasis is the most common cause of colorectal cancer death and is diagnosed with increasing frequency, presenting a pressing clinical need [1]. The liver is a fertile metastatic soil due to a massively parallelled sinusoidal structure, high blood flow, and tolerogenic microenvironment [2]. By the time they are clinically apparent, CRLM achieve an advanced evolutionary state driven by interaction between mutational and epigenetic events and selective pressures afforded by the various tissue environments of the colon, blood, lymphatics and liver [3, 4]. A major component of metastatic evolution is immune evasion [5, 6], which serves as a barrier to successful CRLM treatment using immunotherapeutic approaches.

From a clinical perspective, the presence of CRLM limits efficacy to immunotherapeutics such as immune checkpoint monoclonal antibodies (ICBs) [7]. This is particularly the case for MSS-CRLM which do not display radiological response or overall survival benefit when challenged with either ICB alone, or in combination with novel therapeutics [8, 9]. Conversely, the small proportion of CRLM patients (7.5%) who harbour microsatellite unstable (MSI) CRLM display exquisite sensitivity to ICB [10], which has subsequently become the standard of care for this patient cohort. MSI CRLM display an immune hot, inflamed microenvironment with greater T-cell infiltration when compared to MSS CRLM, indicating that efficient exclusion of T-cells may in part underly the insensitivity of MSS CRLM to ICB. Understanding the mechanisms through which MSS-CRLM exclude T-cells may offer the opportunity to re-engineer the CRLM microenvironment to an inflamed, ICB sensitive phenotype.

Several studies have investigated CRLM immune evasion using sc-RNAseq [11–16], yet sample disaggregation generates small cell numbers, loss of rare, fragile populations and inability to understand spatial context and structure; features that have provided biological insights in CRC [17, 18]. This has hampered understanding of how CRLM co-opt the liver to gain and maintain a survival advantage. Spatial resolution is particularly important for investigating the biologically important region between the CRLM and the liver, which cannot be adequately profiled using disaggregated tissue. Although partially addressed in spatial transcriptomic studies [16, 19], until recently [20], these were not at single cell level, requiring deconvolution for cell composition inference.

Here, we address these shortcomings by combining a human whole-tumour CRLM perfusion platform enabling real-time assessment of spatial T-cell extravasation alongside single-cell protein-based and transcriptomic analysis of large areas of CRLM and background liver, using multiscale spatial techniques (single cell, neighbourhood, topological). We demonstrate impaired T-cell extravasation within the CRLM, alongside evidence of endothelial anergy specifically within CRLM in addition to CD4 T-cell exhaustion in the peri-tumoural liver. Our spatial observations provide a platform for subsequent mechanistic studies to determine whether reversal of CRLM endothelial anergy might improve T-cell infiltration within MSS tumours, with a clinical aim of sensitising to currently available immunotherapies.

## Methods

### Tissues and ethics

All tissues were retrieved from patients undergoing surgical resection of liver metastatic colorectal cancer within the Department of Hepatobiliary Surgery, Oxford, UK and Department of Hepatobiliary Surgery, Liverpool, UK collected under ethics numbers 22/SC/0429 and 21/YH/0206. The clinical details are shown in supplementary Tables 1–3.

### Testing T-cell extravasation in-vivo

#### Normothermic liver perfusion

Surgical resection proceeded in a way as to limit warm ischaemia to < 10 min. Hemi-livers were flushed with 1 L ice-cold Custodiol^®^ with 10,000 IU of unfractionated heparin (5 IU/mL, Panpharma, Luitré, France) until the liver blanched completely and cannulated. Meanwhile, the OrganOx *metra*^®^ was primed with 3 units of O Rh negative packed red blood cells (Cambridge Biosciences, Cambridge, UK) in 500–1000 mL Gelofusine, cycled through the device for pO2 and pCO2 conditioning until optimal temperature (37 °C) and perfusate pH (7.3–7.4) were reached using sodium bicarbonate (B. Braun Medical ltd., Hessen, Germany). Meropenem (500 mg, Consilient Health, Dublin, Ireland) and 10,000 IU heparin were added to the perfusate.

The hemi-liver was onboarded and perfusion started. Throughout perfusion, the following were infused; 0.5 g epoprostanol sodium (Flolan, MercuryPharma, London, UK), 8 µg/hr, 25,000 IU heparin, 833 IU/hr, 5.6 g sodium taurocholate (OrganOx, Oxford, UK), 187 mg/hr, and 200 IU fast-acting insulin (Actrapid, Novo Nordisk, Bagsvaerd, Denmark) titrated to perfusate glucose concentration. Nutriflex parenteral nutrition solution (B. Braun Medical ltd., Kronberg, Germany) was used, dependent on glucose concentration. Arterial and venous flows were regulated autonomously (*metra*^®^) and integrated in-line arterial blood monitoring (Terumo CDI 550; Terumo, 58 Tokyo, Japan) provided O_2_ and CO_2_ partial pressure and pH readings. Arterial blood gas measurements, haematocrit and haemoglobin levels were recorded 4-hourly (i-STAT 1, Abbott, Illinois, USA) and liver biochemistry 8-hourly (Piccolo Xpress, Abbott, Illinois, USA).

At experimental termination, livers were perfused with a further 3 L Custodiol^®^ to remove circulating lymphocytes, before perfusion with 500mls Lycopersicon Esculentum (Tomato Lectin, Sigma Aldrich) to a final concentration of 2 µg/ml.

#### Protein Imaging, QC and segmentation

Multiplexed immunofluorescence (mIF) was performed on formalin-fixed, paraffin-embedded (FFPE) sections using Cell DIVE™ (2500HS, Leica Microsystems, Germany) following standard immunohistochemistry techniques (see supplementary methods). Image stacks from mIF imaging cycles were computationally registered using DAPI stains.

#### Cell-DIVE image QC, segmentation and cell phenotyping

Raw image tiles were stitched and merged (ASHLAR [21]) yielding composite images with autofluorescence subtraction (MCMICRO) [22]. Single-cell segmentation was conducted using Mesmer [23], comprising nuclear and composite membrane/cytoplasmic signals (CK19, αSMA, CD31, CD3, CD4, CD8, CD68) and markers then quantified per-cell using the ark-analysis framework [24]. Arcsinh-transformed marker intensity values derived from the whole-cell segmentation were used as inputs for cell gating with thresholds independently determined per marker, per image (SCIMAP, gate_finder [25]) with cell type annotation performed using hierarchical phenotyping (SCIMAP phenotype_cells [25]). Cells with a nuclear: cell ratio > 1 and those expressing biologically mutually exclusive markers were excluded. Relevant scripts found at: https://github.com/ShihongWu/multiplexed-image-preprocessing.git.

#### CosMx image QC and segmentation

RNA QC was carried out as per NanoString guidelines. Cells with < 50 transcripts or large segmented areas (> 40,000 μm²) were excluded. FOVs with reduced signal or biased gene expression profiles were excluded. Segmentation was performed using Nanostrings Spatial Informatics Portal (SIP) based on the CellPose algorithm [26] refinement through visualisation.

1,930,656 cells, across 1,845 FOVs, were retained into a single dataset with batch effects corrected using harmony (v1.2.3) [27].

### CosMx normalisation and phenotyping

Target counts were normalised by dividing the total counts of each gene within a cell by the total counts of all genes in that same cell and then multiplying by the average total counts per cell across the entire dataset. UMAP was performed using normalised and square root transformed expression values for all genes. Supervised clustering was carried out using the InSituType package (v2.0) using the reference cell profiles provided by Bruker [28]. Validation was performed through examination of protein and marker gene expression and the spatial tissue distribution of the cells. Subset-level clustering was performed on pre-filtered expression data containing only the relevant cell types. Further unsupervised clustering was performed using InSituType to identify transcriptionally distinct groups, which were subsequently annotated based on marker gene expression. Sensitivity analysis was performed for various analyses through removal of the top and bottom 5% of observations based on gene expression to account for outlier bias and by removing a randomly allocated 10% of the datapoints to ensure robustness.

### CosMx-specific analyses

#### Region assignment

A Seurat object was constructed using the expression matrix, annotated cell identities, and corresponding metadata using the Seurat package (v5.3.0) [29]. Tumour, peritumour, and healthy regions were defined on a slide-wise basis based on the Euclidean distance of each cell from the nearest cancer cell. Cells < 50 μm from malignant cell A/B were designated ‘tumour’, within 50–500 μm as ‘peritumour’, and > 500 μm as ‘background liver’.

#### Gene signature scores

Gene signature were calculated using Seurat’s ‘AddModuleScore’ method. Control genes were randomly sampled from 24 expression bins based on average expression levels. Per-cell scores were then computed as the average expression of the signature genes minus that of the control genes, averaged across 25 control sets to correct for differences in baseline expression levels. The Hypoxia gene expression score used in Fig. 7N is developed from reference [30].

#### Distance-signature score gradients

Signature scores were modelled as a smooth function of the Euclidean distance from the nearest tumour cell. For each slide, as well as across all slides combined, generalised additive models were fitted using the ‘bam’ function from the mgcv package (v1.9.1) [31].

#### scRNA-seq processing and analysis

Two independent scRNA-seq datasets were obtained [32, 33]. Following data QC (see supplementary methods), gene expression matrices were log-normalised and scaled using the Seurat functions ‘NormalizeData’ and ‘ScaleData’ and gene signatures quantified using Seurat ‘AddModuleScore’.

### Statistical analysis

Between-region differences for absolute cell and neighbourhood proportions and for selected TDA features were tested at the slide level after averaging across ROIs within each tissue region (CRLM, peri-tumoural liver, background liver) using paired Wilcoxon signed-rank tests with Benjamini–Hochberg false-discovery-rate (FDR < 0.05). Slide-level means are presented as box-and-whisker plots (interquartile range (IQR) boxes, whiskers to 1.5×IQR, median line shown). Throughout figures, p-values represented by *, **, *** and **** are equivalent to < 0.05, < 0.01, < 0.001 and < 0.0001 respectively.

## Results

### Evaluation of T-cell extravasation in perfused human CRLM, peri-tumoural and background liver

**Fig. 1:**
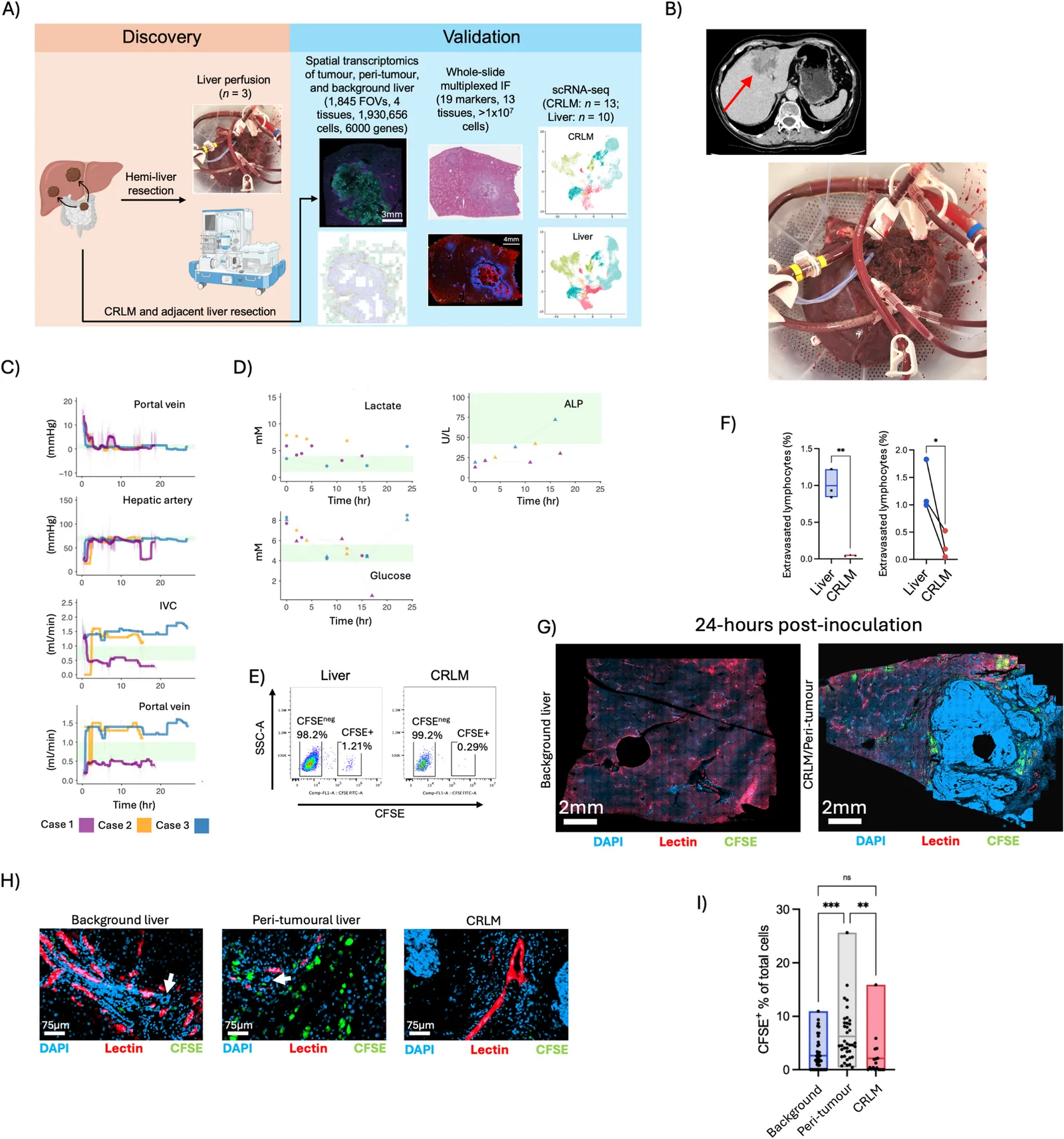
Failure of T-cell extravasation in CRLM. **A** Schematic of the samples used at each stage in the research program. **B** Computed tomography image of CRLM (arrow) and resulting explanted left hemi-liver cannulated to enable normothermic perfusion. **C**-**D** Physiological and biochemical readouts from 3 hemi-livers inoculated with CFSE-labelled T-lymphocytes. Green shades represent physiological ranges. **E** FACS of disaggregated peri-tumoural liver and CRLM showing detection of extravasated CFSE-labelled T-lymphocytes in the liver, with fewer in the CRLM. Samples are taken at the experimental end-point from the experiment demonstrated by the blue curve in (**C**). **F** Quantification of extravasated CFSE^+^ T-lymphocytes in the liver and CRLM across 3 samples from a single perfusion experiment (left) and paired analysis of extravasated T-lymphocyte proportions in liver and CRLM across 3 perfused livers (right). Paired T-test throughout. Samples are taken at the experimental endpoint for each liver demonstrated in (**C**). **G** Whole-slide fluorescence image of background liver taken from an uninvolved vascular segment (above) and the CRLM/peri-tumoural liver (below) at the end of the hemi-liver perfusion experiment. Tomato lectin (red), CFSE and DAPI, nuclear label are shown. **H** High-magnification images taken from (**G**) demonstrating examples of portal triads at the indicated locations, identified morphological through the presence of biliary structures (white arrows) and perfused (Tomato Lectin^+^) vascular channels in keeping hepatic arterial and portal venous branches. **I** Quantification of extravasated CFSE^+^ T-lymphocyte numbers in the background liver (45 ROIs), peri-tumoural liver (38 ROIs) and CRLM (18 ROIs)(T-test) from a single experiment. ALP alkaline phosphatase, SSC-A side scatter

T-lymphocytes are excluded from microsatellite stable (MSS) CRLM [34], which typically demonstrate an immune cold microenvironment, but the exclusion mechanisms are incompletely understood. We hypothesised that impaired lymphocyte extravasation, specifically within CRLM might contribute to T-cell exclusion. The samples used to test and validate this hypothesis include 20 human CRLM specimens with matched background liver, in addition to 23 samples from published single-cell sequencing datasets (Fig. 1A). Initially, we repurposed the OrganOx *metra*^®^ perfusion platform developed for normothermic liver preservation prior to transplantation [35], using this technology instead, to support viability of surgically resected human hemi-livers containing CRLM (Fig. 1B). This provided a novel, entirely human model of the CRLM in their native liver microenvironment, enabling us to study dynamic T-cell extravasation within a cancer and associated peri-tumoural liver for the first time. During a 24-hour period, long enough to compare lymphocyte extravasation into the liver [36] and stroma-rich tumours [37], we perfused specimens (supplementary Table 1 for clinical details) with oxygenated, nutrient-supplemented venous (portal) and arterial blood under physiological pressures and flows (Fig. 1C), maintaining perfusate biochemistry through hepatic function (Fig. 1D).

Within 2 h of perfusion, we inoculated CFSE labelled lymphocytes from ABO-compatible buffy coats into the perfusion fluid. We were able to detect labelled lymphocytes in the perfusate following inoculation using flow cytometry, with the vast majority (> 95%) representing CD4 and CD8 T-cells (Supplementary Fig. 1). By experimental endpoint, we detected significantly fewer extravasated CFSE^+^ cells in the CRLM compared to the liver (Fig. 1E-F). To confirm that FACS-detected lymphocytes had extravasated and ensure adequate CRLM perfusion, we imaged the fluorescent cells alongside infused Tomato Lectin, labelling perfused vasculature and enabling the comparison of perfused regions of background, uninvolved vascular segments, peri-tumoural liver and the CRLM (Fig. 1G-H). Notably, the proportion of extravasated CFSE positive cells was greater within the peri-tumoural liver compared to the CRLM or background liver (Fig. 1H-I), indicating that impaired extravasation contributes to CRLM T-cell exclusion and that circulating lymphocytes primarily extravasate in peri-tumoural liver (Table 1).

**Table 1:** Antibodies used in FACS experiments

Antibody Name	Clone	Fluorophore	Brand
CD45	HI30	BV711	Biolegend
CD3	UCHTI	Pacific blue	Biolegend
CD8	HIT8a	PE	Biolegend
CD4	OKT4	BV605	Biolegend
CD14	HCD14	PeCy5.5	Biolegend
CD16	3G8	PeCy7	Biolegend
CD19	HICB19	APC	Biolegend

### A spatial transcriptomic CRLM liver atlas

**Fig. 2:**
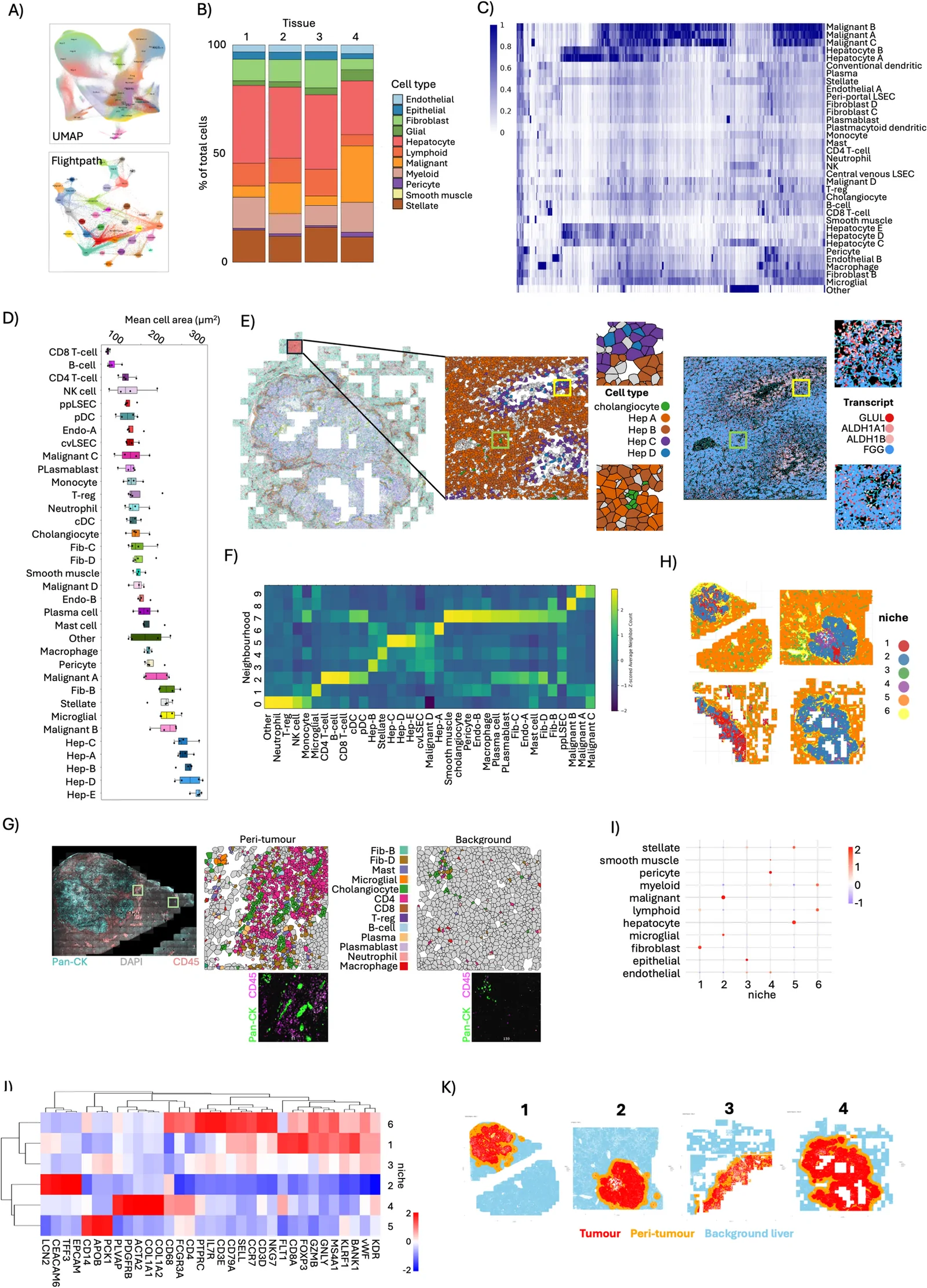
A spatial transcriptomic CRLM liver atlas. **A** UMAP and flightpath plots demonstrating the results of cell clustering within the spatial transcriptomics (CosMx) dataset (enlarged in Supplementary Fig. 3A-B). Data is generated from 4 CRLM patient samples. **B** Stacked bar charts of broad cell types in each of the 4 samples showing the proportion of each cell type across the whole specimen. **C** Heatmap of gene expression for every gene in the CosMx dataset for each cell type. **D** Box-whisker plots of median cell size for each cell phenotype across all 4 samples. **E** Cell types mapped spatially with magnification of the indicated area demonstrating cell segmentation and colour coding of 4 hepatocyte subsets and cholangiocytes (green) demonstrating zonation with regard hepatocyte distribution. Non hepatocyte/cholangiocyte cell types shaded in grey. The same area is then shown to the right, but with colour-coded transcript spots representing the locations of the individual RNA molecules demonstrating hepatic zonation with regards transcript distribution. Given the large size of this sample, FOVs of the peritumour and CRLM were prioritised for transcript imaging, as the maximum FOV number suggested by the manufacturer was reached. **F** Heatmap showing the 10 most frequently occurring cellular neighbourhoods across all samples determined using the KNN method. **G** Whole-slide CosMx immunofluorescence image for the indicated markers (left), with selected CosMx Field of Views (FOVs) from the peri-tumour and background liver demonstrating cell segmentations colour-coded to cell phenotypes. Panel below shows immunofluorescence of the same FOVs demonstrating pan-CK^+^ cholangiocytes whose positions correspond to the cell assignments above. **H** Spatial distribution of 6 cell ‘niches’ across the 4 tissues. **I** Dot plot of broad cell type frequency for each niche. **J** Heatmap demonstrating expression of the indicated genes within each spatial niche. **K** Tissue segmentation masks for each sample demonstrating CRLM, peri-tumoural liver and background liver regions

To investigate the molecular basis of T-cell exclusion, we generated a single-cell spatial transcriptomic atlas from four replacement-type MSS CRLM of matched size and chemotherapy regimen (Supplementary Fig. 2A, Supplementary Table 2). We segmented 1,930,656 cells (35 cell types) (Fig. 2A, Supplementary Fig. 2B-C), ) finding conserved proportions across samples (Fig. 2B and Supplementary Fig. 2D-E) [38]. Cells expressed lineage-specific genes (Fig. 2C and Supplementary Fig. 3C) and demonstrated appropriate phenotype-specific size parameters (Fig. 2D), supporting our segmentation algorithm. The liver showed cell and transcriptome-level zonation across 4 hepatocyte subtypes (Fig. 2D-E and Supplementary Fig. 4A), whilst cholangiocytes expressing hallmark genes *KRT7* and *DEFB1* localised to epithelial structures at the portal triad identified at protein level (Supplementary Fig. 4B). The K-nearest neighbour (KNN) method grouped cell phenotypes into biologically relevant neighbourhoods (cN0-cN9, Fig. 2F). CosMx cN2 contained T and B-cells in addition to plasmacytoid and conventional dendritic cells (pDC and cDC) indicating that it is a potential hub of antigen presentation and adaptive immune response. B-cell and DC presence within cN2 indicates potential antigen presentation and antibody production; features akin to tertiary lymphoid structures (TLS) identified in primary cancers [39].

Although TLS are identified in primary colorectal cancer [40, 41], our spatial segmentation maps did not identify B-cell germinal centres (Fig. 2G), indicating that these are likely lymphoid aggregates rather than mature TLS, supporting that lymphoid aggregates surrounding CRLM rarely represent mature TLS [20]. To investigate how cellular transcriptome changes in different tissue regions, we defined tissue niches from broad cell-type assignments (this is as opposed to the neighbourhood method which considers individual cell type). The resulting niches mapping to their predicted origins in the CRLM or liver (Fig. 2H-I) and bulk transcriptome displayed expression of lineage-specific genes with niche dependency (Fig. 2J). The niches were then combined to generate tumour, peri-tumoural liver and background liver Region of Interest (ROI)s (Fig. 2K). Neighbourhood proportions within different areas of the tissue demonstrated that fibroblast and macrophage neighbourhoods (cN7) are found in both the peri-tumoural liver and CRLM, the lymphoid neighbourhood (cN2) predominantly in the peri-tumoural liver, a neutrophil and Treg-enriched neighbourhood (cN0) in the CRLM, and hepatocyte neighbourhoods (cN3, cN5, cN6) in the peri-tumoural and background liver compartments (Supplementary Fig. 2F). Our analysis pipeline thus provides a detailed visualisation of tissue cellular diversity, highlighting the peri-tumoural liver as an area of significant immune infiltration and potential antigen presentation, consistent with the observations in perfused livers where CFSE-labelled lymphocytes extravasated predominantly within the peri-tumoural liver.

### Endothelial anergy in CRLM endothelium

**Fig. 3:**
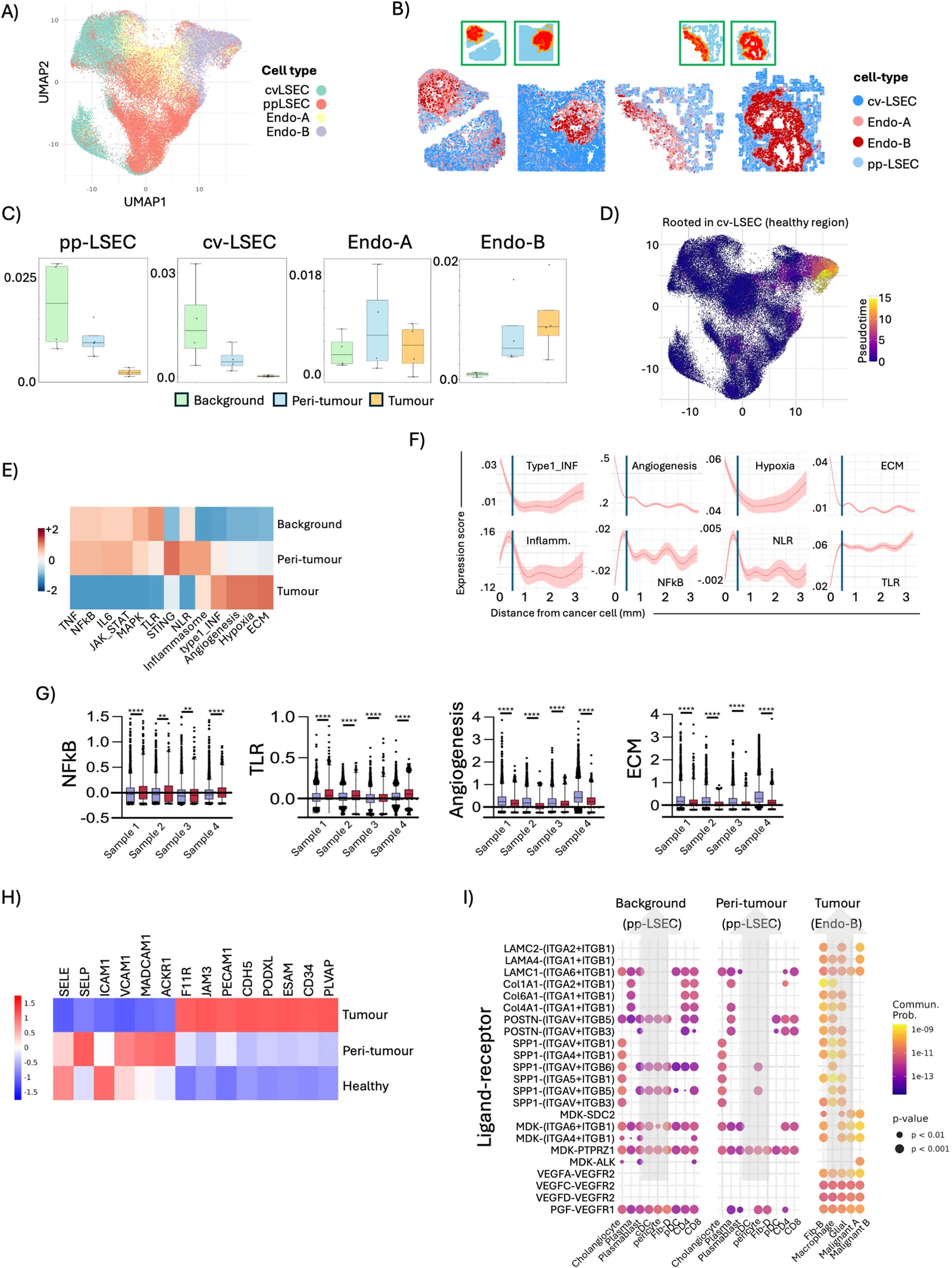
Endothelial anergy in CRLM endothelia. **A** UMAP of endothelial subsets pooled and batch-corrected across all 4 patient samples. **B** Spatial map of the 4 tissues showing the position of the 4 different endothelial cell types. **C** Box-whisker of each endothelial subtype across the indicated regions demonstrating > 2000 endothelial cells per bar. **D** Pseudotime trajectory analysis (Monocle3) of the 4 endothelial populations rooted from the cv-LSEC specifically within the healthy liver pooled and batch corrected across all 4 patients. **E** Heatmap of the indicated transcriptional signatures of endothelial cells within the different tissue regions. **F** Graphs showing transcriptional signature expression (y-axis) in endothelial cells with distance from the nearest malignant cell (x-axis) in mm. The blue vertical line indicates the distance boundary between the peri-tumour and background liver (i.e. 500 μm). Dark curves represent mean and shaded regions around the curve represent the confidence interval. Gene expression score is averaged for each cell across all tissues. **G** Box-whisker plots of the indicated gene expression score for endothelial cells within the CRLM (purple) and peri-tumoural liver (red) for each of the 4 patient samples. Each dot represents a unique endothelial cell and the boxes represent the 95th percentile with the central line representing the median value. **H** Heatmap of expression of selected adhesion molecule genes in all endothelial cells within the indicated tissue regions. **I** Bubble plot of ligand-receptor interactions with different endothelial subtypes within specific tissue locations as the receiver cell (top), ligand-receptor pairs on the y-axis and emitting cell types on the x-axis. Grey arrows show the direction of signalling. Colour and size of dots represent communication strength (relative probability) and p-value is calculated using a random permutation test. Data is generated by pooling transcriptional data across all 4 patient samples

As endothelia regulate T-cell entry into tissues in response to inflammation, we reasoned that differences in endothelial inflammatory signalling in the CRLM and peri-tumoural liver may underlie differences in lymphocyte extravasation and accumulation at these sites. We defined 4 endothelial cell subsets across all samples, including central venous (cv-) and peri-portal (pp-) liver sinusoidal endothelia (LSECs) (Fig. 3A) in the background liver (Fig. 3B-C) and two further endothelial populations; endothelial B (Endo-B) predominantly in the CRLM and peri-tumoural liver and Endo-A distributed throughout the tissue regions (Fig. 3C). As well as demonstrating spatial specialisation, endothelial subtypes are defined by specific transcriptional patterns (Supplementary Fig. 5A). Pseudotime analysis indicated that Endo-B arises from LSECs, with Endo-A an intermediary state (Fig. 3D and Supplementary Fig. 5B), supporting that replacement CRLM co-opt LSEC populations and direct their differentiation to a pro-tumorigenic phenotype as a source of angiogenic vasculature [42, 43]. Intra-tumoural endothelia expressed ECM interaction, hypoxia and angiogenic pathways, whilst peri-tumoural endothelia expressed TNF, NFkB, STING, nod-like receptor (NLR) and inflammasome signatures (Fig. 3E) indicating a response to cellular damage. This was apparent when using distance as a continuous variable, where endothelial inflammation peaked in the peri-tumoural region (Fig. 3F). These changes were consistent across all 4 tissues, as the CRLM endothelial displayed consistently elevated angiogenesis and ECM signalling compared with the peri-tumoural endothelia, whist the latter displayed significantly higher NFkB and TLR signalling (Fig. 3G). These changes were also robust to outlier effects in terms of transcriptional score (removal or highest and lowest 5% (Supplementary Table 4) and spatial location (changing the CRLM-peri-tumoural border by 10% (Supplementary Table 5). Endothelia therefore shift from a non-inflamed state in the normal liver to a state of inflammation in the peri-tumoural liver and a return to non-inflamed state in the tumour. Toll-like receptor (TLR) signalling remains high in the background liver, in keeping with the LSECs role in pathogen and toxin scavenging [44].

**Fig. 4:**
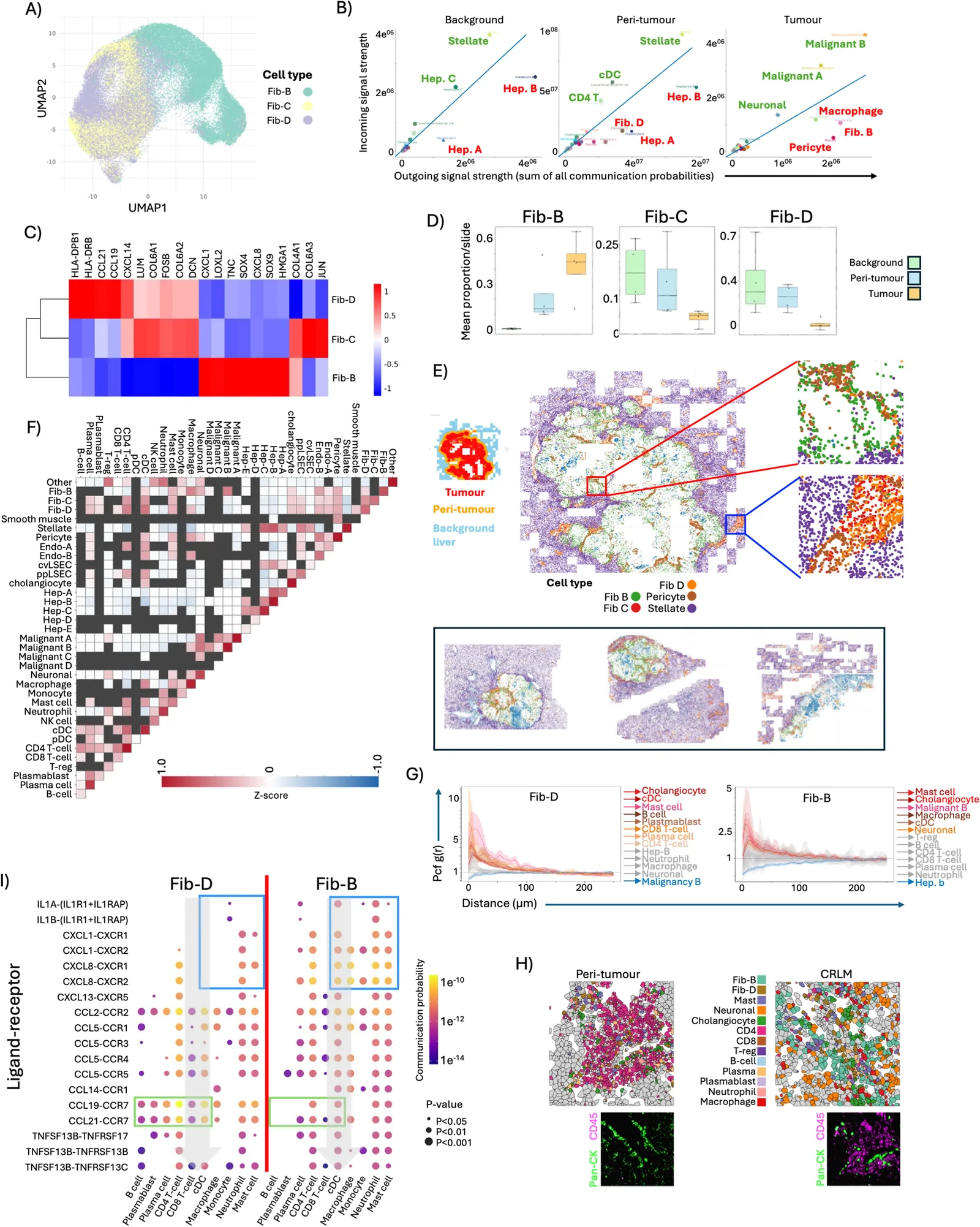
Fibroblast plasticity in CRLM and background liver. **A** UMAP of fibroblasts divided into 3 subtypes across the CosMx dataset. **B** Incoming and outgoing signalling strengths based on cumulative ligand/receptor expression separated based on tissue region and with strongest incoming/outgoing signalling cell types highlighted. Axes indicate the summation of all incoming or outgoing communication probabilities [46]. Cell types in red are net outgoing signallers and those in green are net signalling receivers. The blue line indicates perfect balance in outgoing and incoming communication probability. Cell types in the bottom left of each graph play lesser roles in incoming/outgoing signalling. **C** Heatmap of selected genes from the 3 fibroblast populations. **D** Box-whisker plots of the different fibroblast subtypes in the indicated tissue regions. Given small sample size (4 per condition), statistical difference not formally tested. **E** Whole tissue spatial map of stromal cells with tissue segmentations shown (left) for reference. Higher magnification (right) demonstrates tumour stroma (top) and portal triad in background liver (bottom). Box below demonstrates fibroblast assignments across the remaining 3 tissues within the CosMx dataset. **F** SCIMAP single cell-cell spatial proximity plot showing the likelihood of the indicated cell pairs residing within the ten nearest neighbours of each other. Red indicates spatially correlated and blue represents spatial exclusion. **G** Pair correlation function (PCF) plots describing the ratio of the observed density of cell pairs g(r) (y-axis), at distance r (x-axis) to the expected density under complete spatial randomness. Displaying Fib-D (left) and Fib-B (right) as the index cell. Curves are coloured red (statistically significant spatial association) grey (no association – random distribution) and blues (statistically significant spatial exclusion). Lines indicate means with 95% CI shaded. CI excluding 1 up to at least 100 μm is considered significant. **H** Example cell segmentation masks taken from portal triad identified within the peritumoral liver (left) and CRLM (right) regions. Cholangiocytes are identified in each plot, but with a significantly altered immune-stromal infiltrate inclusive of, Fib-B and macrophages in the latter. **I** Ligand-receptor bubble plot of interactions with Fib-D (left) or Fib-B (right) as the emitter of ligands and various cell types as the receivers (x-axis). Colour and size of dots represent communication strength (relative probability) and p-value is calculated using a random permutation test. Plots limited to the peritumour and CRLM for Fib-D and Fib-B respectively

Inflammation drives adhesion molecule expression in endothelia, and T-cell adhesion molecules *SELP*, *VCAM1*, *MADCAM1* and *ACKR1* were specifically downregulated in CRLM endothelia relative to those in the peri-tumoural liver (Fig. 3H). Conversely, expression of endothelial cell-cell interaction and barrier maintenance genes *JAM1* (*F11R*), *PECAM1*, *CDH5*, *PODXL*, and *PLVAP* were highest in CRLM endothelia (Fig. 3H). By combining 2 publicly available scRNAseq datasets (E-MTAB-12022 and dbGAP identifier phs001818.v3.p1), we also identified higher adhesion molecule expression and upregulation of damage response pathways in liver endothelia compared to those in the CRLM (Supplementary Fig. 5C-E). Ligand-receptor analysis of endothelia segregated to tissue region demonstrated that a specific fibroblast subset fibroblast-B (Fib-B, see Fig. 4), macrophages and cancer cells communicate with Endo-B through ECM basement membrane laminins and collagens (*COL4* and *COL6*), whilst driving angiogenesis through *VEGF*,* PGF*,* MDK* and *SPP1* ligands (Fig. 3I), a feature lacking in endothelia within the background and peri-tumoural liver. Lack of a basement membrane is a recognised feature of the hepatic LSEC population, and our findings indicate progressive anergy and capillarisation of the endothelial tree from the peri-tumoural liver to the CRLM, with potential endothelial-ECM interactions which may provide a further barrier to effective T-cell extravasation [45]. Transcriptional anergy demonstrated here provides a potential molecular explanation for the failed T-cell extravasation observed in the perfusion platform (Fig. 1). This warrants further mechanistic validation to determine whether endothelial anergy is a driver of reduced T-cell extravasation in CRLM and if it is possible to reverse endothelial anergy as a means of overcoming T-cell exclusion.

### Fibroblasts subsets coordinate specific immune niches in CRLM and peri-tumoural liver

CosMx neighbourhood analysis indicated maintenance of a fibroblast-rich neighbourhood (cN7) from the peri-tumoural liver to the CRLM (Fig. 2F and Supplementary Fig. 2E). We identified 3 fibroblast populations (Fib. B-D), with Fib-C and Fib-D demonstrating transcriptional similarity (Fig. 4A). Fib-D and Fib-B displayed strong outgoing signalling capacity in the peri-tumoural liver and CRLM respectively, with neither contributing to signalling in the background liver, indicating a spatial component to fibroblast specialisation and that fibroblasts are key regulators of both the CRLM and peri-tumoural microenvironments (Fig. 4B). Fib-B expressed neutrophil chemo-attractants *CXCL1* and *CXCL8*, ECM remodelling genes *TNC* and *LOXL2* and transcription factors *SOX9* and *HMGA1*, which promote activation and fibrosis in cancer and chronic inflammatory states [47–50]. Fib-C expressed collagen and elastin fibril and ECM modifying genes *Col14a1*,* LUM*,* Col6a1*,* Col6a2*,* Col6a3* and *DCN* and Fib-D expressed genes involved in TLS formation including *CCL21 *[51], *CCL19 *[20] (Fig. 4C). Fib-D also expressed HLA Class II genes, indicating capability to present antigen to CD4 T-cells; a feature identified in cancer-associated fibroblasts at other sites [52, 53].

Fib-B represented the greatest fibroblast proportion in CRLM, with Fib-C and Fib-D in the peri-tumoural and background liver, but rarely in CRLM (Fig. 4D-E) indicating that Fib-D phenotypic loss accompanies and may be mechanistically linked to failure of lymphoid aggregate maintenance in CRLM (Fig. 7, below). Other stromal cells (e.g. stellate) were confined to background liver, with pericytes in both CRLM and liver (Fig. 4E) and these spatial findings were broadly similar across the 4 tissues (Fig. 4E). Fib-D were spatially correlated with pp-LSECs and cholangiocytes indicating residence within the portal triad (Fig. 4F-G), but also with plasma cells, cDCs, CD4 and CD8 T-cells (Fig. 4H), and interaction over longer distances with B-cells (Fig. 4G). Conversely, Fib-B spatially correlated with cancer cells, monocytes and macrophages, and were spatially inversely correlated with both T-reg and CD4 T-cells (Fig. 4G). Both Fib-B and Fib-D showed strong spatial association with cholangiocytes (Fig. 4G) which were present in the peri-tumoural liver and the CRLM (Fig. 4H). Replacement CRLM thus co-opt and replace the surrounding liver, leaving the biliary tree intact, but altering its immune composition from one of immune activation and exhaustion, to myeloid cell infiltration (Fig. 4H). The identification of biliary epithelium within the CRLM serves as a point of reference indicating the position that the portal triad would have taken up before the CRLM grew out into the peri-tumoural liver. Comparison of cellular composition in the peri-biliary positions of CRLM and peri-tumoural liver therefore enables us to understanding of how the CRLM has shaped the immune-stromal phenotype to drive tumour growth into the surrounding liver.

From a cell-cell signalling perspective, Fib-D expressed key mediators of TLS formation to neighbourhood cells including *CCL19*,* CCL21* and *CXCL13* (Fig. 4I), whilst Fib-B expressed macrophage recruiting factors *CXCL1*,* CXCL8*,* CCL2*,* CCL5* and IL1 (Fig. 4I). Fib-D communicated extensively with B- and Plasma cells, with Fib-B displaying a complete lack of such communication (Fig. 4I) indicating that the CRLM becomes devoid of a fibroblast population potentially capable of supporting adaptive anti-tumour immunity and antibody production; key features of functional TLS at cancer sites outside of the liver [39, 54].

### Protein-level spatial analysis of T-cells in CRLM

**Fig. 5:**
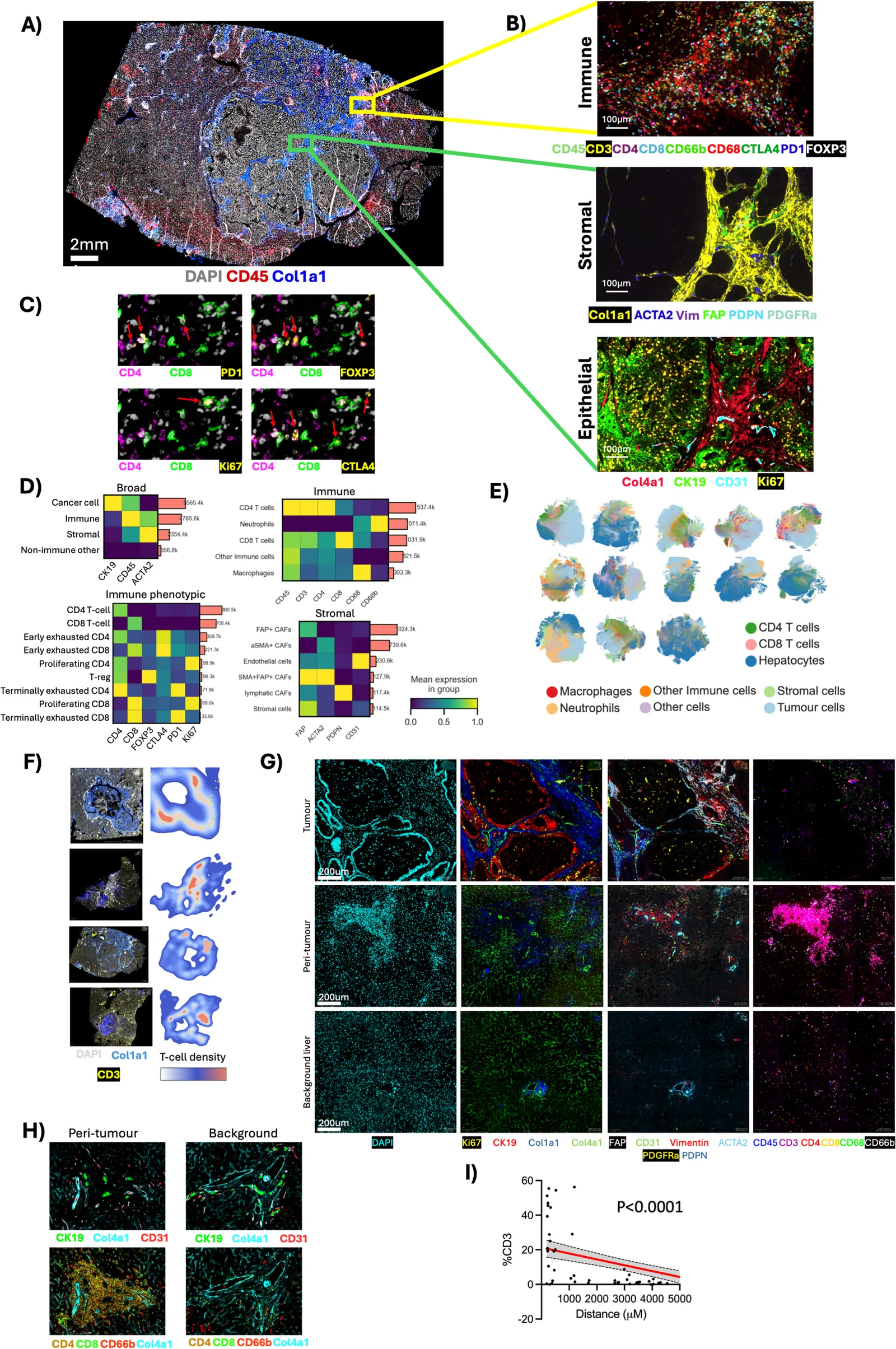
Spatial evidence of T-cell exclusion from the CRLM to the peri-tumoural liver. **A** Whole-slide multiplexed immunofluorescence (mIF) of a CRLM and background liver from a larger dataset of 13 images obtained using the Cell DIVE platform (Leica) with inset (**B**) demonstrating antibody panels at higher magnification. **C** Identification of early exhausted (CTLA4^+^), late exhausted (PD1+), proliferating (Ki67^+^) CD4 and CD8 T-cell populations and CD4^+^/FOXP3^+^ T-regs. Red arrows indicate double positive cells. **D** Heatmap for cellular phenotyping strategy demonstrating marker expression and the number of each cell type identified across the 13-slide dataset (right of each heatmap). **E** Uniform manifold approximation and projection (UMAP)s of broad cell phenotypes for each sample demonstrating degree of variation in cell proportions for each sample. **F** mIF for the indicated markers and matched T-cell spatial heatmaps from 4 representative samples of the 13-sample dataset demonstrating exclusion of T-cells from the tumour (indicated by Col1a1 staining). **G** mIF for the indicated makers from representative areas of the tumour, peri-tumoural liver and background liver showing organised lymphocytic infiltrates in the peri-tumoural liver which are lacking from the other tissue regions. **H** High magnification comparison of the portal triad in the peri-tumoural and background liver demonstrating the CD4-rich lymphoid aggregate in the former. **I** Graph of correlation between CD3 count (proportion of total segmented cells) per portal triad, y-axis and distance of portal triad from the liver-tumour interface in microns (x-axis) with data derived from all samples within the dataset. *P*-value reported for Pearsons correlation coefficient

To spatially validate the lymphocyte exclusion identified in our perfused liver experiments, we profiled 13 MSS whole-slide CRLM and background liver samples (Fig. 5A and Supplementary Fig. 6A, Table 3), simultaneously visualising 19 proteins (Fig. 5B and Supplementary Fig. 6B) and enabling the identification of T-cell phenotypic subsets covering exhaustion (PD1/CTLA4) and activation states (Ki67^+^) (Fig. 5C). To streamline image quality control, segmentation and phenotyping, we developed a bespoke spatial analysis framework [55], enabling the definition of 23 cell types on marker expression (Fig. 5D). Across all tissues, we found higher numbers of CD4 than CD8 T-cells (Fig. 5D), validating our CosMx data (Supplementary Fig. 2D). There was variation in phenotyped cell proportion across tissues (Fig. 5E), likely secondary to different proportions of liver and cancer across specimens (Supplementary Fig. 6A).

Morphologically, T-cells were excluded from CRLM (Fig. 5F) and resided in peri-tumoural aggregates at the portal triad (Fig. 5G), again corroborating the CosMx data, where T-cell aggregates were identified adjacent to biliary epithelium in the peri-tumoural liver. These aggregates contained exhausted and, to a lesser extent, proliferating T-cells (Supplementary Fig. 7A-B). In the Cell-DIVE images, the portal triad is identified by Col1a1 deposition, CK19^+^ biliary epithelia and hepatic arteries lined by a Col4a1-rich basement membrane. This is morphologically distinct from the hepatic sinusoidal vasculature lined by Col4a1 alone (Fig. 5G). Notably, we identified organised lymphoid aggregates in the peri-tumoural liver but not within either the background liver or CRLM (Fig. 5G H), whilst peri-portal lymphoid numbers decreased with distance of the portal triad from the CRLM (Fig. 5I). Together, this data demonstrates lymphocyte exclusion from CRLM and indicates the presence of a CRLM-specific immune response in the peri-tumoural liver that dissipates with distance from the tumour-liver border (Table 2).

**Table 2:** Antibodies used in Cell-DIVE mIF

Antibody Name	Clone	Host Species	Isotype	Cat #	Brand	Concentration
FAP	F1A4G	Rabbit	IgG	81192SF	Cell Signalling Technology	5 µg/mL
CD31	89C2	Mouse	IgG1	3528 S	Cell Signalling Technology	5 µg/mL
Ki67	SP6	Rabbit	IgG	ab281847	Abcam	10 µg/mL
CD45	RM1007	Rabbit	IgG	ab281586	Abcam	10 µg/mL
ACTA2	1A4	Mouse	IgG2a	ab184675	Abcam	5 µg/mL
CD4	EPR6855	Rabbit	IgG	ab133616	Abcam	10 µg/mL
CD45	RM1007	Rabbit	IgG	ab281586	Abcam	10 µg/mL
COL4A1	1042	Mouse	IgG2b	51-9871-82	Invitrogen	10 µg/mL
CD3	SP162	Human	IgG	ab245731	Abcam	10 µg/mL
CD66b	G10F5	Mouse	IgM	305,110	Biolegend	10 µg/mL
FOXP3	D2W8E	Rabbit	IgG	74,816	Cell Signalling Technology	5 µg/mL
CTLA4	CAL49	Rabbit	IgG	ab237712	Abcam	10 µg/mL
PD1	EPR4877(2)	Rabbit	IgG	ab137132	Abcam	10 µg/mL
PDPN	D2-40	Mouse	IgG1	916,606	Biolegend	10 µg/mL
CK19	EPR1579Y	Rabbit	IgG	ab232566	Abcam	5 µg/mL
PDGFRa	D1E1E	Rabbit	IgG	32,641	Cell Signalling Technology	5 µg/mL
COL1A1	E8F4L	Rabbit	IgG	51-9871-82	Cell Signalling Technology	10 µg/mL
Vimentin	EPR3776	Rabbit	IgG	ab185030	Abcam	5 µg/mL
CD68	EPR20545	Rabbit	IgG	ab277276	Abcam	10 µg/mL
CD8	C8/144B	Mouse	IgG1	372,902	Santa Cruz	10 µg/mL

### Spatial disruption of T-cell aggregates in CRLM

**Fig. 6:**
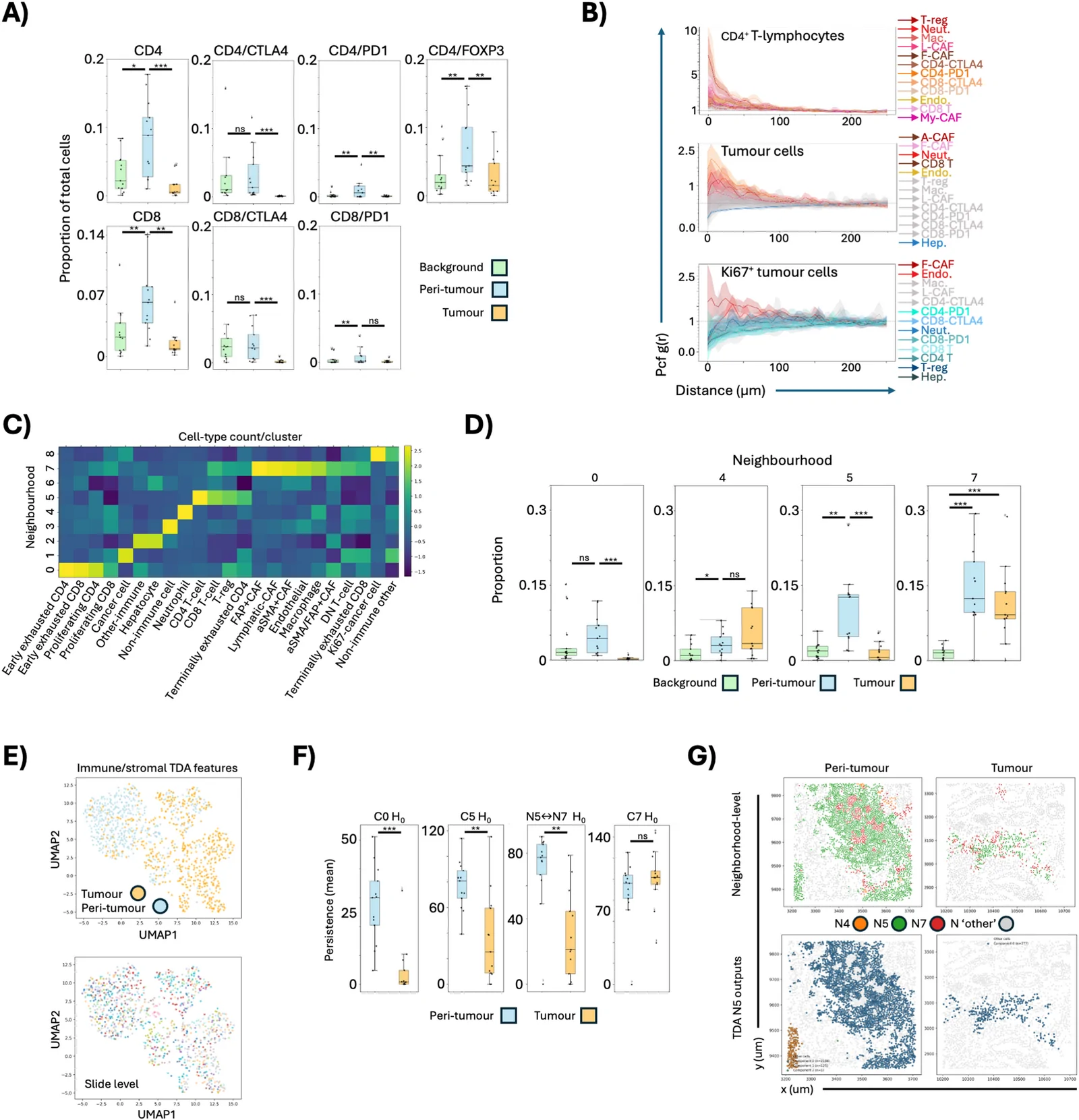
Spatial mapping and topological analysis of immune-stromal neighbourhoods. **A** Box-whisker plots of CD4 and CD8 T-cell subsets in the CRLM, peri-tumoural liver (liver tissue ≤ 500 μm of the CRLM edge) and background liver (liver tissue > 500 μm from the CRLM edge). Individual sample medians from multiple ROIs within each sample represented by points (i.e. 13 points per bar), central line represents median, and error bars interquartile range (IQR). Analysed using paired Wilcoxon signed-rank tests at the slide level adjusted for false discovery (Hochberg FDR) [58]. **B** PCF plots with settings as per Fig. 4G, but using CD4^+^ T-lymphocytes (top), non-proliferating cancer cells (middle) and proliferating cancer cells (bottom) as the index cells. Graphs are generated from averages across all samples (see methods). **C** Heatmap showing the 9 most frequently occurring cellular neighbourhoods across all 13 samples determined using the KNN method. **D** Box-whisker plots of N0, N4, N5 and N7 frequency in the indicated tissue regions across all 13 samples. Presentation and analysis as per (**A**). **E** UMAP of N0, N5 and N7 TDA features selected using Random Forest classifiers applied to vectorised TDA curves describing the structural arrangement of these neighbourhoods in the tumour (orange) and peri-tumoural liver (blue). Lower pane demonstrates the slide that each point from the UMAP was derived, with the 13 samples colour coded demonstrating lack of batch effect and supporting that the tissue region distinguishing TDA features are common between samples. **F** Box-whisker plots of the indicated TDA feature persistence in the CRLM and peri-tumoural liver. Presentation and analysis as per (**A**). **G** Spatial map of the peri-tumour (left) and CRLM (right) colour coded by neighbourhood (top) and N5 TDA component (bottom) highlighting breakup of T-cell enriched structures within the CRLM

We next segmented tissues into ‘CRLM’ (bounded by CK19^+^ cells), ‘peri-tumoural’ liver (cells ≤ 500 μm from the CRLM) and ‘background liver’ (cells > 500 μm from the CRLM) matching that generated in the CosMx analysis and enabling robust comparison of tissue compartments across samples and platforms. This enables us to compare cell-cell spatial interactions on a per-cell level to understand how these differ across the three tissue compartments. CD4, and to a lesser extent, CD8 T-cells including early (CTLA4^+^), terminally exhausted (PD1^+^) and proliferating phenotypes, were most prevalent in the peri-tumoural liver, indicating that it is the primary site of immune activation and exhaustion (Fig. 6A) [56, 57]. CD4 T-cells demonstrated significant spatial interaction with other immune cell types across cell-cell signalling distances (< 100 μm) (Fig. 6B). Cancer cells, conversely, demonstrated spatial association with fibroblasts and endothelia, with proliferating cancer cells demonstrating strong exclusion of all T-cell subsets (Fig. 6B). Proliferating cancer cells therefore reside in protected, immune-excluded niches and lymphocytes form organised communities in the peri-tumoural liver, excluded from the CRLM.

K-Nearest Neighbour (KNN) analysis identified cellular neighbourhoods of early exhausted and proliferating CD4 T-cells (Neighbourhood 0, N0), and of CD4 T-cells, CD8 T-cells, terminally exhausted CD4 T-cells and T-regs (N5) (Fig. 6C). N0 and N5 existed in the peri-tumoural liver and were almost completely absent from the CRLM (Fig. 6D). Fibroblast, endothelia, macrophage, double-negative T-cell and terminally exhausted CD8 T-cell clusters (N7) appeared at equal proportion in peri-tumoural liver and CRLM (Fig. 6D). Broadly, this validates the spatial transcriptomic observations and demonstrates that the peri-tumoural liver is a hub of immune activation and exhaustion, with the outgrowing CRLM retaining stromal elements of the peri-tumoural liver inclusive of neutrophils, macrophages and CAFs, but selectively excluding its lymphocytic component. Finally, we applied topological data analysis (TDA) to N0, N5 and N7, allowing us to map the degree to which inter-related stromal and immune structures change from the peri-tumorual liver into the CRLM. TDA can distinguish the frequency of disconnected ‘islands’ and their spatial longevity prior to merging with other islands (H_0_). This approach identifies organisational features between cellular neighbourhoods that are of potential biological importance. We used a random forest approach to identify features from the resulting TDA curves describing neighbourhood topology. This enabled us to distinguish the peri-tumour and CRLM with high cross-validated accuracy (72.7 ± 7.4%) (Fig. 6E and Supplementary Fig. 8A), demonstrating that the CRLM and peri-tumoural liver display unique topology with regards stromal and immune neighbourhoods. The top 30 TDA features were dominated by H_0_ descriptors from N0, N5, and stromal/myeloid rich interactions (e.g. N5↔N7) (Supplementary Fig. 8A-B), with each significantly elevated in the peri-tumour (Fig. 6F) indicating that immune-stromal spatial relationships are maintained in the pari-tumoural liver and lost in the CRLM. Conversely, N7 (fibroblast/macrophage) persistence was equivalent in the CRLM and peri-tumour (Fig. 6F). These findings support a model whereby the CRLM maintains myeloid and stromal structures, whilst structurally fragmenting exhausted lymphoid aggregates as it grows into and co-opts the peri-tumoural liver (Fig. 6G). This phenocopies the CosMx data, where fibroblast-cholangiocyte spatial relationships were maintained across the peri-tumoural liver and CRLM, with loss of lymphoid populations and their structural integrity in the latter.

### CD4 T-cell dysfunction in the CRLM and peri-tumoural liver

**Fig. 7:**
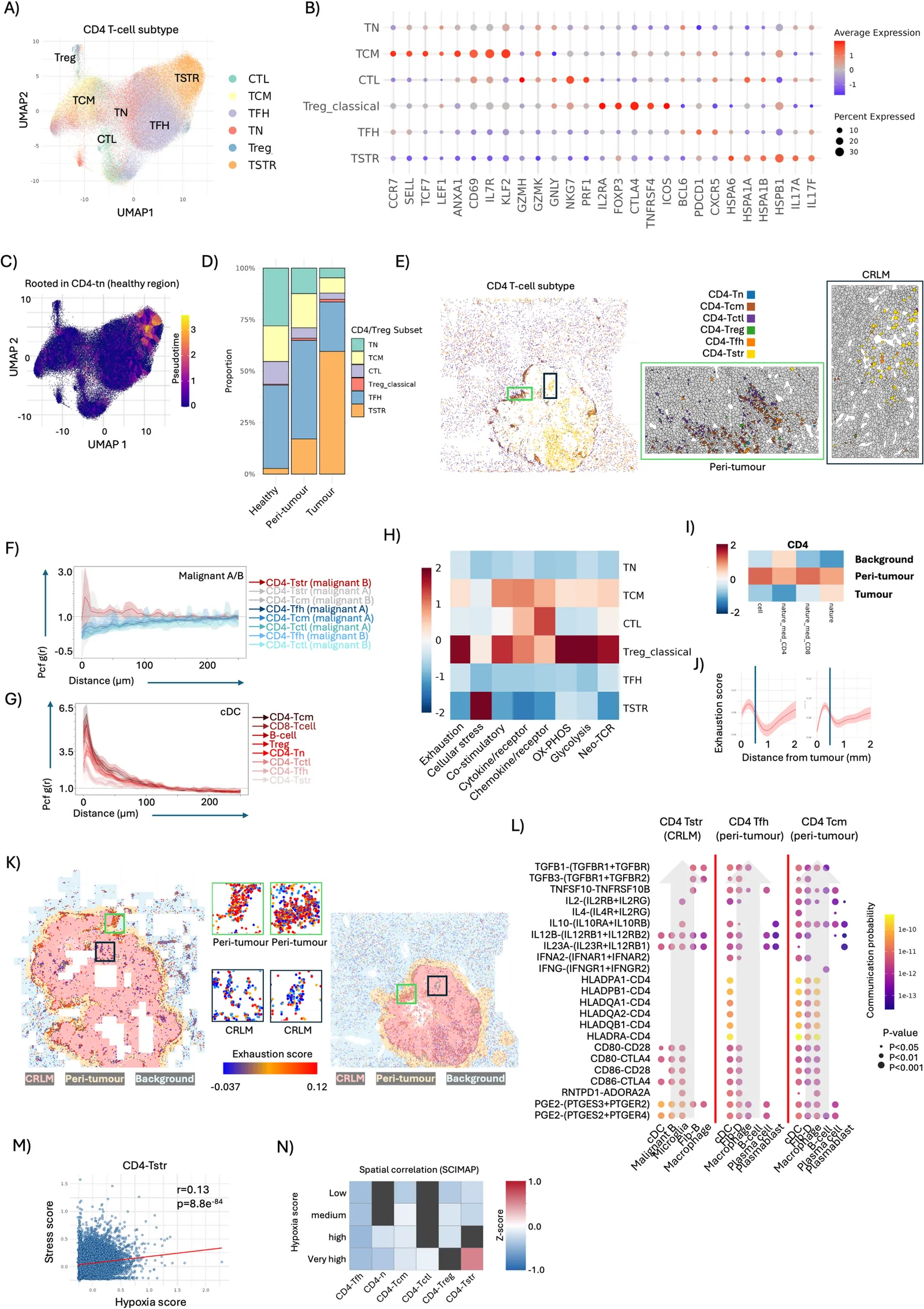
CD4 T-cell dysfunction in the CRLM and peri-tumoural liver.**A** UMAP of CD4 T-cell subsets across all 4 samples following batch correction. **B** Dot plot of marker expression displaying hallmark genes for each CD4 T-cell subset. **C** Pseudotime trajectory analysis of CD4 T-cell subsets across the 4 pooled samples. **D** Stacked bar plot of the proportion of different CD4 T-cells subsets in the indicated tissue regions with proportions represented as an average across the 4 samples. **E** Spatial map with CD4 T-cell subsets colour-coded see Fig. 2(K) for CRLM/peritumour/background boundaries. **F**-**G** PCF plots showing the spatial relationships between CD4 T-cell subsets and malignant A/B cells (**F**) and cDC (**G**) with settings as per Fig. 4G. H) Heatmap of the expression of different transcriptional pathways (x-axis) in each of the CD4 T-cell subsets. **I** Heatmap of the indicated T-cell exhaustion signatures (x-axis) in CD4 T-cells from the three tissue regions. Exhaustion signatures were from references [65] (Cell) [66], (Nature Medicine) and [67] (Nature). **J** Graphs showing exhaustion signature expression in CD4 T-cells with distance from the nearest malignant cell (x-axis) in mm. Settings as per Fig. 3F. The signature source is indicated above each graph. **K** Spatial map of CD4 T-cells in 2 of the samples colour-coded based on enrichment of the Cell exhaustion signature score from (**I**). The CRLM, peri-tumour and background liver are overlayed in pink, yellow and blue respectively. Inset high-magnification boxes show heavily exhausted cells in the peri-tumoural liver (top boxes) and lower levels of exhaustion in the CRLM (lower boxes). **L** Bubble plot of ligand-receptor interactions with settings as per Fig. 3H, showing signalling from different cell types (x-axis) converging on the indicated CD4 T-cell subsets (top). Grey arrows show the direction of signalling. **M** Scatter plot of transcriptional stress (x-axis) and hypoxia (y-axis) signatures in the CD4-Tstr population. **N** SCIMAP single cell-cell spatial proximity plot showing the likelihood of the different CD4 T-cell subsets residing within the ten nearest neighbours of any cell type from the CosMx dataset based on the cells hypoxic state (expression level of hypoxic gene signature from reference [30]). Red indicates spatially correlated and blue represents spatial exclusion

Our Cell DIVE data identified CD4 T-cell predominance in the peri-tumoural liver, supporting published findings [56, 57]. CD4 T-cells are of increasingly recognised importance in the CRLM TME [56], and display diverse functional repertoires [59–61]. In adoptive T-cell trials, neoantigen-specific CD4 T-cell, but not CD8 T-cell proportions correlate with clinical response, pointing to their role in mediating anti-tumour immunity [62]. Because CD4 T-cells mediate hepatic inflammation [63], maintain TLS through interaction with immune and stromal cells [64], and were the predominant immune population in the peri-tumoural liver, we decided to return to the spatial transcriptome dataset to understand CD4 subset spatial biology in greater depth. Unsupervised clustering of CD4^+^ cells defined 6 new sub-populations (Fig. 7A) including follicular helper (CD4-Tfh) expressing *BCL6*,* PDCD1* and *CXCR5*, cytotoxic (CD4-Ctl) expressing *GZMH* and *NKG7*, central memory (CD4-Tcm) expressing *CCR7*,* KLF2* and *IL7R*, T-regulatory (CD4-Treg) expressing *FOXP3*,* CTLA4* and *TNFRSF4*, stress response state (CD4-Tstr), expressing *HSPA1A*,* HSPA1B*, and *HSPB1* and naïve CD4 T-cells (CD4-Tn) (Fig. 7B). Pseudotime analysis indicated that CD4-Tstr is a terminal subset (Fig. 7C). CD4-Tcm were found at higher number in the peri-tumoural liver (Fig. 7D-E) and were excluded from cancer cells (Fig. 7F), whereas the CD4-Tstr represented the majority of intra-tumoural CD4 T-cells (Fig. 7D and Supplementary Fig. 9A-B) and were the only CD4 subtype spatially correlated with cancer cells (Fig. 7F). Despite variation in the proportion of several CD4 subsets between samples, CD4-Tcm loss and CD4-Tstr gain from the peri-tumour to the CRLM was a common feature across samples (Supplementary Fig. 9B). CD4 T-cells displaying a stress response are identified in lung, breast and renal cancers [60], but not previously in CRLM.

Central memory T-cells are those that respond to previously encountered antigen. In keeping with this, CD4-Tcm showed the strongest spatial correlation with antigen presenting cells such as cDC (Fig. 7G), and upregulation of a neo-TCR transcriptional pathway indicative of response to neoantigen (Fig. 7H) [68]. CD4-Tcm also displayed cytokine expression and upregulation of co-stimulatory molecules but simultaneously displayed high levels of exhaustion (Fig. 7H). The CD4-Tstr subset lacked cytokine expression capability and demonstrated a low TCR score indicating failure to maintain neoantigen reactive CD4 T-cells within the tumour core either through loss of TCR-reactive clones, or through their reversion to a non-TCR reactive subtype. CD4-Tstr did not display high levels of exhaustion and overall, CD4 T-cell exhaustion levels peaked in the peri-tumoural liver (Fig. 7I-K), where CD4-Tcm and CD4-Tfh are found in high numbers. This validates the Cell-DIVE findings and indicates that exhaustion is not a mechanism through which the CRLM drives immune evasion within the tumour core, but that peri-tumoural exhaustion may be brought about through chronic antigen exposure, with CD4 T-cells becoming dysfunctional through alternative pathways in the CRLM, resulting in a cellular stress response. 

Supporting these ideas, we found that antigen presenting cells including cDCs, Fib-D and macrophages interact with CD4-Tcm in the peri-tumorual liver through MHC and co-stimulatory molecules *CD80/CD-86* (Fig. 7L). In the peri-tumoural liver CD4-Tcm receive strong incoming signalling through *IL10*, *TGFb1*, *TGFb3* and *TRAIL* (*TNFSF10*), all of which drive exhaustion and (in the case of *TRAIL*), promote T-cell apoptosis [69]. Conversely, CD4-Tstr receive no MHC signalling and are exposed to prostaglandin (*PGE2*) and IL23A by several different cell types. *PGE2* does not affect T-cell priming, but specifically prevents the expansion of effector T-cell populations within the TME [70] and is a prominent driver of T-cell stress responses [71]. Furthermore, *PGE2* and *IL23A* combine to drive *IL17* expression by CD4 T-cells [72], a cytokine with both anti- and pro-tumourigenic effects expressed by the CD4-Tstr population (Fig. 7B). Hypoxia is also an important driver of the cellular stress response and a prominent feature of the TME. Notably CD4-Tstr cellular stress response was positively correlated with hypoxia signalling on a single cell level (Fig. 7M), and CD4-Tstr cells but not other CD4 populations demonstrated spatial correlation with the most hypoxic cells across all tissue regions (Fig. 7N) indicating that the CD4-Tstr specifically reside within hypoxic microenvironments.

## Discussion

Here, we demonstrate, for the first time, failed T-cell extravasation in human MSS CRLM alongside with endothelial anergy at single-cell spatial resolution. Whilst not providing direct mechanistic evidence that endothelial anergy impairs T-cell extravasation in our perfusion model, our spatial transcriptomics observations indicate that MSS CRLM endothelia are highly anergic, lacking the key molecules required for T-cell extravasation and so potentially represent an important cellular mediator of the T-cell exclusion phenotype in MSS CRLM. This hitherto unrecognised role may explain why immunotherapeutic approaches such as adoptive T-cell therapies, which rely on effective extravasation of injected exogenous cells have broadly failed in this population and potentially in solid tumours more broadly.

Endothelial anergy has previously been identified in endothelia cultured from tumours and normal tissue, with the former displaying lower levels of leukocyte adhesion molecules ICAMs and VCAM [73]. Our findings are an advance on this, as they confirm endothelial anergy in a human tumour setting. Furthermore, we provide direct evidence of impaired extravasation of circulating T-cells into MSS CRLM under physiological conditions. Mechanistic approaches looking to overcome endothelial anergy are now required to investigate whether this is a mechanism through which MSS CRLM exclude T-lymphocytes and if it represents an actionable pathway for clinical translation. Similarly, although we confirmed T-cell exclusion using mIF and spatial transcriptomics, a phenotype well recognised in MSS CRLM, our findings are unable to explain how myeloid subsets including macrophages nonetheless appear capable of entering the MSS CRLM microenvironment. Given that specific macrophage subsets play a pro-tumourigenic role in CRLM, any attempt to increase T-cell extravasation through endothelial anergy reversal would have to simultaneously guard against unwanted macrophage extravasation. Better understanding of the precise relationship between endothelial adhesion molecule expression and immune subset extravasation within the CRLM vascular bed is therefore required before the translational relevance of our findings can be realised.

Anti-angiogenic therapies can overcome endothelial anergy, leading to enhanced endothelial-leukocyte interactions in-vitro [74], indicating that angiogenesis and anergy are part of a shared continuum opposing a state of endothelial inflammation. Our findings support this concept, as we identified activation of angiogenic pathways in the spatial transcriptomics dataset coupled with anergy in the CRLM endothelia driven predominantly by VEGF family members secreted by cancer cells, myeloid cells and fibroblasts through spatial ligand-receptor interaction. Notably, the combination of anti-angiogenic strategies alongside immune checkpoint blockade has demonstrated limited efficacy in MSS CRLM [75]. Our findings indicate that this may be because the exhausted T-cell compartment is spatially separated from the CRLM and that a potentially more fruitful approach may be to combine anti-VEGF with adoptive T-cell approaches (ACT). Given that CAR T-cell extravasation is dependent on endothelial ICAM-1 expression [76], combining ACT with therapeutics that drive endothelial inflammation may enhance ACT uptake into CRLM.

In addition to the demonstration of intra-tumoural endothelial anergy, our data indicate that lymphocytes signposted for extravasation in the peri-tumoural liver through endothelial inflammation and fibroblast-derived chemokine expression, become exhausted before reaching the CRLM. We demonstrate this both using spatial transcriptomics, where we identify different CD4 T-cell phenotypic states which demonstrate spatial segregation and in the mIF dataset where exhausted CD4 T-cells are abundant in the peri-tumoural liver. Exhaustion of T-cells in the peri-tumoural liver might provide a conducive soil for CRLM outgrowth, which it accomplishes through co-option of the hepatic sinusoid [43]; a process we provide supportive evidence for through identification of retained biliary epithelia within the CRLM indicative of portal triad structural maintenance. Identification of intra-tumoural biliary epithelium through mapping of the unique cholangiocyte transcriptome enabled us to show that outgrowing CRLM replace exhausted peri-portal lymphoid aggregates with macrophages, potentially recruited through the secretory activity of plastic fibroblast populations.

The few CD4 T-cells identified within the CRLM are presumably retained from the peri-tumoural liver at their site of extravasation within the portal triad, where they subsequently undergo transcriptional changes driven by the cellular microenvironment of the CRLM, or migrate into established CRLM from the surrounding liver. We identified scarce intra-tumoural CD4 T-cells within hypoxic niches where they displayed a stress response, impaired T-cell receptor and cytokine activity rather than evidence of an exhaustion signature. This implies that lymphocyte exhaustion is a peri-tumoural phenomenon pre-dating CRLM outgrowth into the liver parenchyma. Future approaches utilising the perfusion platform should look to test these various hypotheses through transcriptional profiling of T-cells at various stages of extravasation and in response to therapeutics designed to reverse endothelial anergy. Reversal of the T-cell stress response also provides a therapeutic avenue of interest, as this may negate the need to alter endothelial anergy given that the stressed T-cell population already resides within the tumour microenvironment.

Importantly, our focus on large areas of tissue in both the perfusion and spatial biology experiments will have helped overcome aspects of within-tumour heterogeneity that could potentially act as significant confounders. Similarly, by limiting our analysis to replacement-type, MSS CRLM treated with neoadjuvant chemotherapy, we focus on the most common clinical scenario, the most pressing clinical need, and helped to reduce between-patient confounding in view of the small sample size. The benefits of this process are borne out in several parts of the analysis where we demonstrate robust and consistent representation of cell type composition across the 4 transcriptionally profiled tissues, in addition to the mIF series. Similarly, the key findings of both CRLM-specific endothelial anergy and peri-tumoural T-cell exhaustion were consistent across all tissues. Observations from the perfusion platform also appeared consistent across specimens and this was further aided by the ability to access patient-matched non-involved liver tissue as a comparator for T-cell extravasation. Nonetheless, the small sample size is a major limitation of our work and we recognise that profiling a larger cohort will be essential to confirm the presence of the identified cell states across patient subsets and ultimately, to correlate their presence and spatial transcriptional state with clinical outcome.

## Conclusions

In summary, coupling of a novel in vivo human tumour perfusion system with a large single-cell spatial transcriptomic dataset from whole-slide specimens validated through mIF has provided a wealth of findings of relevance for the therapeutic targeting of immune evasion in CRLM. Our findings represent the most detailed spatial analysis of the CRLM microenvironment and background liver to date, providing a roadmap for future mechanistic studies aiming to sensitise to immunotherapeutic approaches.

## Supplementary Information

Supplementary Material 1. Supplementary Figure 1: Gating strategy for flow cytometry. A) FACS plots of perfusion samples post red cell lysis demonstrating expression of CFSE in a proportion of the circulating immune compartment. Subsequent plots are pre-gated on the CFSE positive population and demonstrate presence of B-cell, CD4/CD8 T-cell and NK cell markers. The perfusate sample was taken 5 minutes following lymphocyte inoculation. B) FACS plots of disaggregated tissue samples taken at the experimental endpoint demonstrating gating strategy and showing that the CFSE expressing population that extravasates within the liver consists of lymphocytes expressing CD3, CD4 and CD8 with a lesser population expressing CD19. Supplementary Figure 2: CosMx platform QC. A) Immunofluorescence for indicated markers from CosMx platform on the 4 tissues used to generate the spatial transcriptomic atlas. B) Example of a flagged FOV exhibiting diminished total counts or biased gene expression profiles leading to exclusion. C) Violin plots demonstrating the number of reads per FOV for each tissue (top) and the number of reads per cell (bottom) across the entire CosMx dataset. D) Stacked bargraphs showing the proportion of each cell type across the 4 specimens within the CosMx dataset. Red dots represent corresponding group mean. E) Stacked bargraphs showing the proportion of each neighbourhood defined within the CosMx dataset (Fig. 2F) across the three tissue regions. Supplementary Figure 3: Gene expression characteristics of single cell transcriptomic atlas. A) Flightpath plot enlarged image from Fig. 2(A). B) UMAP cell clustering of CosMx data enlarged from Fig. 2(A). C) Heatmap of selected hallmark genes defining each cell type in the CosMx dataset. Supplementary Figure 4: Validation of phenotyping strategy in hepatocytes and cholangiocytes. A) Spatial cell phenotyping from CosMx data with hepatocyte subsets colour-coded based on clustering from Fig. 2A. Hepatocytes A and B are predominantly in a peri-portal location (sinusoidal zone 1), whilst hepatocytes C and D are found nearer the central vein (sinusoidal zone 3). Cholangiocytes demonstrated in green. Subsequent panels demonstrate CosMx-detected transcript positions for genes known to regulate peri-portal (red) or pericentral (blue) functions superimposed on cholangiocyte segmentations (green). These data spatially validate the phenotypic assignment of hepatocyte subsets. B) CosMx immunofluorescence for the indicated markers showing positive pan-cytokeratin staining in cell clusters within the background liver. Subsequent panels show CosMx phenotyped cholangiocytes (brown) alongside detections of the canonical cholangiocyte markers KRT7, PDGFD, SCD5 and DEFB1, all of which are enriched in the cholangiocyte segmentations. Supplementary Figure 5: Single cell sequencing of pooled CRLM and background liver validates CRLM endothelial anergy and reduced adhesion molecule expression. A) Heatmap of top 30 differentially expressed genes in each endothelial cell subtype. B) Pseudotime plots for endothelial cells rooted in the indicated endothelial subtype and spatial location. C) UMAP clustering of CRLM and background liver samples from two batch corrected single cell sequencing datasets with cell types colour coded. D) Violin plots of endothelial adhesion molecule expression for the indicated genes in the CRLM and background liver. E) Heatmap of the indicated gene expression signatures in endothelial cells from the CRLM and background liver using the same signatures as those in Fig. 3. Supplementary Figure 6: mIF dataset. A) H&E and matched mIF of the Cell-DIVE dataset stained for CK19 (red) and collagen 1 (blue). B) Whole slide image of CRLM (tumour) and background liver showing DAPI and matched H&E with tumour positions marked. The distribution of each marker is demonstrated below. Supplementary Figure 7: Organised lymphoid aggregates in peri-tumoural liver contain exhausted lymphocytes. A) Multiplexed image of lymphoid aggregate in the peri-tumoural liver demonstrating the predominance of CD4 T-cells over CD8. This is a portal triad, as evidenced by spatially matched multiplex demonstrating the presence of CK19^+^ biliary epithelium surrounded by Col4a1 and ACTA2+ fibroblasts. B) Multiplexed image of peri-tumoural liver and CRLM microenvironment for the indicated markers demonstrating organisation of lymphocytes and expression of PD1 in the former. C) Multiplexed image demonstrating ROI generation used to define portal triad. Biliary tree (CK19), portal vein (CD31) and hepatic artery (CD31 surrounded by ACTA2^+^ myofibroblasts) branches are identified. Resultant ROI demonstrated by yellow border. Supplementary Figure 8: Feature selection for Cell DIVE TDA. A) Ranked bar plot of TDA features discriminating peritumour and tumour regions, as identified by random forest classifier feature importance (mean decrease in impurity, MDI). Each bar corresponds to a single TDA feature, derived from persistent homology calculations applied to single and pairwise neighbourhood clusters (e.g., NC0, NC4, NC5, NC7, and their pairings), and represents its mean importance across cross-validation folds. The top 30 features, as ranked by average MDI, are indicated above a dashed red line. B) Volcano plot comparing the distribution of the same TDA features between regions. For each feature, the x-axis gives the difference in slide-level medians (tumour minus peritumour). Y-axis is -log10 of the FDR-adjusted p-value from a paired Wilcoxon signed-rank test. Each point represents one TDA feature, with the top 30 features highlighted and labelled, and those not passing FDR significance are noted as non-significant in the legend. Supplementary Figure 9: T-cell exhaustion and phenotypic states across tissues. A) Spatial maps of the 6 CD4 T-cell subsets. High-magnification regions within the CRLM and peri-tumoural liver demonstrate the differences in CD4 T-cell subsets in these locations, with the CD4 Tstr subset identified within the CRLM shown at higher magnification below through cell segmentation masks. B) Distribution of the indicated CD4 T-cell subtypes across the 4 specimens and within the 3 different tissue regions.

Supplementary Material 2.

Supplementary Material 3.

## Data Availability

CosMx spatial transcriptomic data generated in this study can be obtained from the NCBI GEO database with accession number GSE312757. No new algorithm was developed for this study. Analyses were performed using pre-existing tools.
