# Supplementary material for: Evidence of endothelial anergy and T-cell exclusion in microsatellite stable colorectal liver metastases revealed through human hemi-liver perfusion and spatial transcriptomics
Source: Mol Cancer. 2026 Jul 20;25:244. doi: 10.1186/s12943-026-02732-4 (PMC13673828; doi:10.1186/s12943-026-02732-4)
Supplement: Supplementary file 1 [file 12943_2026_2732_MOESM1_ESM.docx]

**Supplementary Figures**

**Supplementary Figure 1. Gating strategy for flow cytometry. A)** FACS plots of perfusion samples post red cell lysis demonstrating expression of CFSE in a proportion of the circulating immune compartment. Subsequent plots are pre-gated on the CFSE positive population and demonstrate presence of B-cell, CD4/CD8 T-cell and NK cell markers. The perfusate sample was taken 5 minutes following lymphocyte inoculation. **B)** FACS plots of disaggregated tissue samples taken at the experimental endpoint demonstrating gating strategy and showing that the CFSE expressing population that extravasates within the liver consists of lymphocytes expressing CD3, CD4 and CD8 with a lesser population expressing CD19.

**Supplementary Figure 2. CosMx platform QC. A)** Immunofluorescence for indicated markers from CosMx platform on the 4 tissues used to generate the spatial transcriptomic atlas. **B)** Example of a flagged FOV exhibiting diminished total counts or biased gene expression profiles leading to exclusion. **C)** Violin plots demonstrating the number of reads per FOV for each tissue (top) and the number of reads per cell (bottom) across the entire CosMx dataset. **D)** Stacked bargraphs showing the proportion of each cell type across the 4 specimens within the CosMx dataset. Red dots represent corresponding group mean. **E)** Stacked bargraphs showing the proportion of each neighbourhood defined within the CosMx dataset (Figure 2F) across the three tissue regions.

**s**

**Supplementary Figure 3. Gene expression characteristics of single cell transcriptomic atlas. A)** Flightpath plot enlarged image from Figure 2(A). **B)** UMAP cell clustering of CosMx data enlarged from Figure 2(A). **C)** Heatmap of selected hallmark genes defining each cell type in the CosMx dataset.

**Supplementary Figure 4. Validation of phenotyping strategy in hepatocytes and cholangiocytes. A)** Spatial cell phenotyping from CosMx data with hepatocyte subsets colour-coded based on clustering from figure 2A. Hepatocytes A and B are predominantly in a peri-portal location (sinusoidal zone 1), whilst hepatocytes C and D are found nearer the central vein (sinusoidal zone 3). Cholangiocytes demonstrated in green. Subsequent panels demonstrate CosMx-detected transcript positions for genes known to regulate peri-portal (red) or pericentral (blue) functions superimposed on cholangiocyte segmentations (green). These data spatially validate the phenotypic assignment of hepatocyte subsets. **B)** CosMx immunofluorescence for the indicated markers showing positive pan-cytokeratin staining in cell clusters within the background liver. Subsequent panels show CosMx phenotyped cholangiocytes (brown) alongside detections of the canonical cholangiocyte markers KRT7, PDGFD, SCD5 and DEFB1, all of which are enriched in the cholangiocyte segmentations.

**Supplementary Figure 5. Single cell sequencing of pooled CRLM and background liver validates CRLM endothelial anergy and reduced adhesion molecule expression.** **A)** Heatmap of top 30 differentially expressed genes in each endothelial cell subtype. **B)** Pseudotime plots for endothelial cells rooted in the indicated endothelial subtype and spatial location. **C)** UMAP clustering of CRLM and background liver samples from two batch corrected single cell sequencing datasets with cell types colour coded. **D)** Violin plots of endothelial adhesion molecule expression for the indicated genes in the CRLM and background liver. **E)** Heatmap of the indicated gene expression signatures in endothelial cells from the CRLM and background liver using the same signatures as those in Figure 3.

**Supplementary Figure 6. mIF dataset.** A) H&E and matched mIF of the Cell-DIVE dataset stained for CK19 (red) and collagen 1 (blue). B) Whole slide image of CRLM (tumour) and background liver showing DAPI and matched H&E with tumour positions marked. The distribution of each marker is demonstrated below.

**Supplementary Figure 7. Organised lymphoid aggregates in peri-tumoural liver contain exhausted lymphocytes.** A) Multiplexed image of lymphoid aggregate in the peri-tumoural liver demonstrating the predominance of CD4 T-cells over CD8. This is a portal triad, as evidenced by spatially matched multiplex demonstrating the presence of CK19^+^ biliary epithelium surrounded by Col4a1 and ACTA2^+^ fibroblasts. B) Multiplexed image of peri-tumoural liver and CRLM microenvironment for the indicated markers demonstrating organisation of lymphocytes and expression of PD1 in the former. C) Multiplexed image demonstrating ROI generation used to define portal triad. Biliary tree (CK19), portal vein (CD31) and hepatic artery (CD31 surrounded by ACTA2^+^ myofibroblasts) branches are identified. Resultant ROI demonstrated by yellow border.

**Supplementary Figure 8. Feature selection for Cell DIVE TDA. A)** Ranked bar plot of TDA features discriminating peritumour and tumour regions, as identified by random forest classifier feature importance (mean decrease in impurity, MDI). Each bar corresponds to a single TDA feature, derived from persistent homology calculations applied to single and pairwise neighbourhood clusters (e.g., NC0, NC4, NC5, NC7, and their pairings), and represents its mean importance across cross-validation folds. The top 30 features, as ranked by average MDI, are indicated above a dashed red line. **B)** Volcano plot comparing the distribution of the same TDA features between regions. For each feature, the x-axis gives the difference in slide-level medians (tumour minus peritumour). Y-axis is -log10 of the FDR-adjusted p-value from a paired Wilcoxon signed-rank test. Each point represents one TDA feature, with the top 30 features highlighted and labelled, and those not passing FDR significance are noted as non-significant in the legend.

sssss

**Supplementary Figure 9. T-cell exhaustion and phenotypic states across tissues. A)** Spatial maps of the 6 CD4 T-cell subsets. High-magnification regions within the CRLM and peri-tumoural liver demonstrate the differences in CD4 T-cell subsets in these locations, with the CD4 Tstr subset identified within the CRLM shown at higher magnification below through cell segmentation masks. **B)** Distribution of the indicated CD4 T-cell subtypes across the 4 specimens and within the 3 different tissue regions.
