# Supplementary material for: Evidence of endothelial anergy and T-cell exclusion in microsatellite stable colorectal liver metastases revealed through human hemi-liver perfusion and spatial transcriptomics
Source: Mol Cancer. 2026 Jul 20;25:244. doi: 10.1186/s12943-026-02732-4 (PMC13673828; doi:10.1186/s12943-026-02732-4)
Supplement: Supplementary file 2 [file 12943_2026_2732_MOESM3_ESM.docx]

**Supplementary methods.**

**Hemi-liver perfusion methods**

**PBMC isolation**

PBMCs were obtained from ABO-compatible whole-blood leukocyte cones (NHS Blood and Transfusion Service) using SepMate PBMC tubes (STEMCELL Technologies, UK), red cell lysis buffer (Qiagen, Germany) and DPBS wash. The PBMC pellet was stained with CellTrace™ CFSE (Invitrogen, UK) before wash and resuspension.

**FACS analysis**

Liver and tumour excisional biopsies were dissociated (Tumour Dissociation Kit, Miltenyi Biotech, UK) and the cell suspension strained (70µM). Whole blood samples were centrifuged and treated with red cell lysis buffer. Cells were stained with Live/Dead Fixable Near-IR dead cell stain (Thermofisher Scientific, UK) and fixed with 10% formalin (Merck). Antibodies used for cell-type detection are shown in table 2. Flow data was acquired on a Cytoflex Flow Cytometer (Beckman Coulter, UK) and analysed using FlowJo v10.10.0 (BD Biosciences).

**Multiplexed IF Analysis**

**mIF staining protocol**

Following deparaffinization and two-step antigen retrieval (citrate buffer pH 6.0 then Tris-EDTA buffer pH 9.0), slides were permeabilized with 0.05% Triton X-100, then blocked with 1% BSA, 1% normal goat serum and 1% normal donkey serum in PBS containing 0.05% Tween-20. Slides were initially stained with DAPI and imaged across all fluorescence channels to capture tissue-specific autofluorescence. In the first staining round, slides were incubated with three antibodies (table 2) and imaged. All rounds employed directly conjugated primary antibodies targeting up to three markers per cycle, with DAPI included in each round. Whole-slide images were acquired using a motorized widefield fluorescence microscope equipped with a 20×/0.75 NA objective. Fluorescence was collected sequentially in DAPI (~460 nm), FITC (~520 nm), Cy3 (~570 nm) and Cy5 (~670 nm) channels. Exposure times were optimized using single-stained controls and held constant across all samples. Between cycles, fluorophores were inactivated using three consecutive 15-minute incubations in fresh alkaline hydrogen peroxide buffer (3% H₂O₂ in 20 mM NaHCO₃, pH 11.2), protected from light. Slides were re-imaged to confirm complete signal removal and capture new autofluorescence profiles, then de-coverslipped for the next staining cycle. This stain–image–bleach sequence was repeated until the full panel was completed.

**Quantification of CD3 counts in portal triad regions**

Image segmentation and analysis were performed in QuPath. Cell-DIVE images were limited to markers of the portal triad including Col4a1, CD31 and CK19 enabling positive identification of portal triads in the background and peri-tumoural liver. To define the portal triad as a separate ROI, we triangulated the most distant visible triad structures (bile duct, hepatic artery or portal vein). Example of resultant ROIs are demonstrated in Supplementary Figure 7C. CD3-positive cell count was normalised against total cell count within each corresponding ROI and presented as a percentage.

**Spatial Transcriptomics Analysis**

**CosMx tissue staining protocol**

Tissues were prepared and processed on the CosMx Spatial Molecular Imager (Bruker Spatial Biology, USA) as per the CosMx SMI Manual Slide Preparation for RNA Assays and CosMx SMI Instrument User Manual. 5 μm thin FFPE sections were mounted to Leica Bond Plus slides (Leica Biosystems, Richmond, USA) within 2 weeks of imaging and baked overnight at 60°C to increase tissue adherence. After baking, the slides were dewaxed and antigen retrieval carried out using Target Retrieval Solution (Bruker Spatial Biology, USA). Tissues were permeabilised using 3 μg/mL Proteinase K and fiducials applied, followed by post-fixation in 10% neutral buffered formalin (NBF). Tissues were washed with NBF stop buffer and then treated with fresh acetate solution and the slides hybridised with CosMx Human 6K probe mix and incubated overnight^60^.

The slides were then washed with stringent buffer in a water bath twice before incubation with in blocking buffer. This is followed by morphology and segmentation marker staining using CD298/B2M mix (Bruker Spatial Biology, USA) and PanCK/CD45 mix (Bruker Spatial Biology, USA) as recommended in the standard protocol for non-neuronal tissues. Following this, flow cells were prepared using CosMx Flow Cell Assembly Kit and loaded on to the CosMx Spatial Molecular Imager. Configuration C was used for Pre-Bleaching profile, and Configuration E was used for Cell Segmentation profile. Following image acquisition, FOVs were selected and cycling and data collection were initiated. FOVs were specifically selected to cover regions of the CRLM, peri-tumoural and background liver with a limit set at 500 FOVs per tissue.

**Spatial transcript preprocessing and polygon segmentation**

Cell boundaries were reconstructed using CellPoly, which infers polygonal outlines for individual cells based on transcript spatial coordinates to capture cellular morphology and spatial organisation. The resulting segmentation maps were visualised as polygon overlays for representative FOVs.

**Pseudotime Trajectory analysis**

Pseudotime analysis was performed using Monocle3, with trajectories rooted in indicated cell types and tissue regions. Pseudotime values were compared across cell types and tissue regions to characterise cell state transitions.

**Cell-cell communication**

Cell-cell communication was analysed using the CellChat (v2.2.0)^67^. Separate spatial CellChat objects were generated for tumour, peritumour, and healthy regions. Communication probabilities were calculated using the ‘computeCommunProb’ and ‘computeCommunProbPathway’ function, with an interaction range of 100 µm and a contact range of 10 µm to account for diffusion-mediated and direct cell-cell interactions, respectively.

**Single-cell sequencing analysis**

**scRNA-seq processing and analysis**

QC was performed according to the parameters reported in the respective studies removing low-quality cells characterised by low gene counts or high mitochondrial gene expression. Datasets were normalised with the Seurat (v5.3.0) function 'SCTransform'^64^. Processed samples were merged, and batch effects corrected using Harmony (v1.2.3)^62^. The corrected embeddings were used for UMAP visualisation. Unsupervised clustering was performed using 'FindNeighbors' and 'FindClusters' to identify distinct cell clusters and cell types annotated based on marker genes.

**Broad spatial analysis methods**

**Neighbourhood Generation**

A radius-based QC step filtered spatially isolated cells likely representing segmentation noise or debris. Cells with <3 cells within a fixed Euclidean radius of 100 centroids (65um after scaling) were removed ensuring that retained cells had >2 neighbours. Following Schürch *et al*.^13^, per-cell neighbourhoods were constructed independently in each dataset (Cell DIVE and CosMx). Within each ROI, we used scikit-learn’s NearestNeighbors (v1.6.1)^70^ to find for every cell, its 10 closest neighbours and counted how many of each cell type appeared among those neighbours. This count vector defined the cell’s neighbourhood composition, and stacking these vectors yielded the per-cell neighbourhood feature matrix^13^. Slide-level batch effects were corrected using ComBat (scanpy v1.11.3), while preserving the region structure (covariates=region)^71,72^. Batch-corrected matrices were clustered with K-means, with the number of clusters selected by an elbow analysis of within-cluster inertia across k=1−15. Cluster identities were defined by the z-scored mean frequency of each cell type among member neighbourhoods and used to annotate the per-cell metadata. Relative enrichment and depletion of cell types in each CN compared to their global prevalence across the tissue was imputed. Missing or ill-posed values (NaN or infinite) were replaced with zeros to ensure numerical stability.

**UMAP generation**

At the ROI level, we computed the proportion of cells assigned to each neighbourhood cluster, applied ComBat (scanpy) and standardisation (StandardScaler, scikit-learn), and embedded using UMAP (umap-learn v0.5.7; n_neighbors=50, min_dist=0.5, spread=2.0, random_state=42)^73,74^. Two-dimensional embeddings were visualised with ROIs coloured by tissue region and by slide to assess region separation and residual batch structure.

**SCIMAP cell-cell interaction**

To quantify short-range spatial interactions independent of tissue region, we pooled all ROIs across the CosMx cohort and computed 10-nearest-neighbour (10-NN) interactions per slide with scimap (v2.3.4)^59^. Per-cell coordinates were loaded into an AnnData object with imageid=slide_ID. We then ran sm.tl.spatial_interaction with method="knn", knn=10, permutation=1000, and pval_method="zscore", yielding slide-level, directional Z-scores and permutation p-values for every ordered pair *i→j* (enrichment if Z>0, avoidance if Z<0).

Directional outputs were exported to long format, then symmetrised within each slide by averaging *i→j* and *j→i* scores (and the corresponding per-slide p-values).

Across slides, the cohort summary for each unordered cell-type pair used the mean symmetrised Z-score, and a conservative aggregate p-value given by the maximum across slides. Pairs were displayed in non-grey colours only if the per-slide (symmetrised) p-value was <0.05 on every slide. Final figures show a lower-triangular heatmap ordered by a curated cell-type list, with a centred diverging colour map (saturating at ±1 Z), and non-significant cells were masked in grey.

**Higher-order spatial cell-cell interaction analyses**

**Cross-Pair Correlation Function (PCF)**

The Muspan spatial-statistics package (v1.1.1) computed cross-pair correlation functions (cross-PCF), gij(r), for every ROI in each dataset^75^. ROI-level curves were then pooled within each cohort (Cell DIVE and CosMx) to generate cohort-level summaries. For each ROI, per-cell coordinates (µm) were registered in a muspan domain alongside categorical cell_type labels, and boundaries were estimated with an alpha-shape hull (α=95 μm) with a 10 µm boundary exclusion for edge correction. In a small number of ROIs (n = 3), where the α = 95 μm hull was suboptimal, we re-estimated the boundary with α = 120 μm and recomputed PCFs for those ROIs only. We exhaustively enumerated all ordered cell-type pairs (including self–self) and evaluated cross-PCFs on a common distance grid (0–250 µm; 5 µm steps; 10 µm annuli). Pairs undefined in a given ROI (one or both cell types absent) were written as NaN and omitted from aggregation using NaN-aware means and standard deviations. Outputs from each ROI were recorded under undirected pair labels (“A ⟷ B”), zero-distance artefacts were suppressed (exclude_zero=True), and ROI computations were parallelised per slide. For cohort summaries, curves were aligned to the common grid and averaged only across ROIs in which the pair occurred. Uncertainty was visualised with approximate 95% confidence bands using the normal approximation μ±1.96 σ/√n, where n is the number of contributing ROIs per pair. For display, we generated overlay plots of pre-specified cell-type pairs, showing cohort-level mean gij(r) curves with the 95% confidence bands and the complete randomness reference line at gij(r) = 1. The same workflow was applied independently to the Cell DIVE and CosMx cohorts, yielding whole-cohort cross-PCF summaries that indicate cell-cell clustering (gij(r)>1), randomness (gij(r)=1), and exclusion (gij(r)<1) across spatial scales.

**Topographical Data Analysis (TDA)**

TDA was applied to neighbourhood-labelled point clouds in each ROI within the Cell DIVE dataset. Two modes were analysed: single-cluster (centroids of all cells from one neighbourhood cluster) and pairwise-cluster (centroids of all cells from both members of an unordered cluster pair, pooled into a single point set). Vietoris–Rips filtrations were computed with muspan (v1.1.1) using a maximum distance of 707 µm (the diagonal of a 500 µm × 500 µm ROI), and persistent homology was evaluated up to dimension 1 to obtain H0 (connected components) and H1 (loops) barcodes^76^. Analyses were restricted to clusters or cluster pairs with at least three cells per ROI, and persistence diagrams for each ROI were saved for all valid clusters and pairs in that ROI.

TDA persistence diagrams were vectorised into interpretable features^77^, including means, standard deviations and selected percentiles of births, deaths, lifespans and midpoints, plus persistence entropy. To emphasise biologically salient structure, we additionally derived “significant-bar” features after thresholding at ≥50 µm for H0 ≥10 µm for H1. For H0, the significant-bar count was incremented by one to reflect the number of components present just before merges at the 50 µm threshold. ROIs were retained if at least one targeted cluster or cluster pair exhibited a significant H0 component. The final ROI-level panel (1520 ROIs) comprised significant bar counts for H0 and H1, and the means and standard deviations of significant H0/H1 lifespans and significant H1 birth times. UMAPs were generated using the top features ranked by random-forest importance (mean decrease in impurity; Cell DIVE: N = 30). For hypothesis testing, ROI-level feature values were averaged across ROIs from the same slide within the same tissue region to yield one slide-level value per region per slide.

Random forest feature redundancy was screened using pairwise Spearman correlations and Benjamini–Hochberg adjustment; “strong” correlation was predefined as R2≥0.7 (4.7% of tests), supporting the full selected feature panel for classification^78^. Region discrimination was evaluated with random forests trained on ROI-level TDA features (scikit-learn v1.6.1, 1,000 trees) using grouped five-fold cross-validation by slide^79^. Accuracy is reported as mean ± standard deviation across folds, and features were ranked by mean decrease in impurity.

To visualise the spatial pattern of targeted neighbourhood clusters within individual ROIs, we generated ROI-level scatter plots from the per-cell metadata containing cluster labels. Clusters 0, 4, 5, and 7 were highlighted using distinct colours, while all other clusters were rendered light grey to provide context (matplotlib v3.10.1). To visualise H0 structure at the biologically motivated threshold (50 µm), we produced ROI-level component maps for selected neighbourhood clusters. For a chosen ROI and cluster, cells were subset, and a undirected graph was built by connecting points within 50 µm (scikit-learn v1.6.1, radius_neighbors_graph), and connected components were identified (SciPy v1.15.2, connected_components)^80^. Each component was plotted in a distinct colour with all other cells shown in light grey for context, using equal axis scaling in micrometres.
